# Advanced Fiber Type-Specific Protein Profiles Derived from Adult Murine Skeletal Muscle

**DOI:** 10.3390/proteomes9020028

**Published:** 2021-06-08

**Authors:** Britta Eggers, Karin Schork, Michael Turewicz, Katalin Barkovits, Martin Eisenacher, Rolf Schröder, Christoph S. Clemen, Katrin Marcus

**Affiliations:** 1 Medizinisches Proteom-Center, Medical Faculty, Ruhr-University Bochum, 44801 Bochum, Germany; karin.schork@rub.de (K.S.); michael.turewicz@rub.de (M.T.); katalin.barkovits@rub.de (K.B.); martin.eisenacher@rub.de (M.E.); 2 Medical Proteome Analysis, Center for Protein Diagnostics (PRODI), Ruhr-University Bochum, 44801 Bochum, Germany; 3 Institute of Neuropathology, University Hospital Erlangen, Friedrich-Alexander University Erlangen-Nürnberg, 91054 Erlangen, Germany; rolf.schroeder@uk-erlangen.de; 4 German Aerospace Center, Institute of Aerospace Medicine, 51147 Cologne, Germany; christoph.clemen@uni-koeln.de; 5 Center for Physiology and Pathophysiology, Institute of Vegetative Physiology, Medical Faculty, University of Cologne, 50931 Cologne, Germany

**Keywords:** skeletal muscle, fiber types, proteomics, laser microdissection, neuromuscular disorders

## Abstract

Skeletal muscle is a heterogeneous tissue consisting of blood vessels, connective tissue, and muscle fibers. The last are highly adaptive and can change their molecular composition depending on external and internal factors, such as exercise, age, and disease. Thus, examination of the skeletal muscles at the fiber type level is essential to detect potential alterations. Therefore, we established a protocol in which myosin heavy chain isoform immunolabeled muscle fibers were laser microdissected and separately investigated by mass spectrometry to develop advanced proteomic profiles of all murine skeletal muscle fiber types. All data are available via ProteomeXchange with the identifier PXD025359. Our in-depth mass spectrometric analysis revealed unique fiber type protein profiles, confirming fiber type-specific metabolic properties and revealing a more versatile function of type IIx fibers. Furthermore, we found that multiple myopathy-associated proteins were enriched in type I and IIa fibers. To further optimize the assignment of fiber types based on the protein profile, we developed a hypothesis-free machine-learning approach, identified a discriminative peptide panel, and confirmed our panel using a public data set.

## 1. Introduction

Skeletal muscle is a heterogeneous tissue composed of muscle fibers, connective tissue, nerve fibers, and blood vessels. The individual protein expression pattern of a single muscle fiber is of fundamental interest to improve our current understanding of specific molecular mechanisms, including the selective loss of different fiber subtypes in response to a variety of factors. Fiber types are classically subdivided into slow type I and fast type IIa, IIb, and IIx fibers [1,2]. Type IIb fibers are exclusively expressed in rodents [3]. All fiber types have defined tasks, e.g., a holding function, continuous movements, or abrupt movements, and therefore, they have unique proteomic compositions. Addressing the latter, it is thus crucial to study individual muscle fiber types in order to determine their specific proteomic patterns in response to, e.g., training, aging, denervation, or intrinsic myopathic processes.

In general, three classification approaches are widely used for the distinction of different skeletal muscle fibers: (1) traditional classification based on an ATPase activity staining technique; (2) differentiation via energetic properties. Slow fibers have a high level of oxidative phosphorylation activity, while fast fibers mainly rely on glycolytic events to sustain their energy supply [4,5]. The third and most widely used approach is differentiation via the specific myosin heavy chain (MYH) isoform expression pattern [6,7,8]. Type I fibers (slow) mainly contain myosin-7 (MYH7), type IIa fibers (fast) contain myosin-2 (MYH2), and type IIx fibers (fast) contain myosin-1 (MYH1), whereas myosin-4 (MYH4) is mainly found in type IIb fibers (very fast), as summarized in Table 1 [3,5]. Notably, skeletal muscles are highly dynamic with a remarkable level of plasticity that allows individual fibers to alter their MYH isoform pattern. In addition to pure slow and fast fiber types, there are intermediate forms that contain multiple MYH isoforms [2,3,9,10]. This plasticity allows fast adaptation in response to various conditions, including exercise and training, physical inactivity, aging, and myogenic, neurogenic, inflammatory, and metabolic disease processes [5,11,12,13,14,15,16,17]. For example, aging studies have been associated with selective wasting and a loss of type II fibers [13,18,19,20,21], whereas type I fiber predominance or the selective affection of fast fiber types has been described in a wide variety of primary muscle diseases, e.g., in the group containing myofibrillar myopathies (MFM) and Duchenne muscular dystrophy (DMD) [22,23,24,25,26]. Until now, the associated molecular processes, inducing fiber type shift, have been poorly understood, but knowledge of these processes could lead to novel therapeutic options. Hence, fiber type analysis at the omics level is emerging as a major field of study to determine the selective involvement of fiber types in processes related to age, exercise, and disease.

Thus, further progress in our current understanding of the protein diversity of individual muscle fiber types was provided by transcriptomic and proteomic analyses. For instance, the transcriptome profiles of total muscle tissue in comparison with derived type I and type IIa fibers provided insight into the levels of mitochondrial, cytosolic, and muscle structure coding genes as well as the distinct expression of different protein isoforms, e.g., caveolin, synaptotagmin, and tropomodulin, within the specific fiber subtypes [27]. Later proteomic studies of muscle fiber subtypes succeeded in revealing further specific features such as fiber type-specific protein changes during muscle atrophy [28], different mitochondrial adaptations of the fiber types as well as age-dependent differences in energetic profiles between young and old muscle fibers derived from healthy human subjects [12,13]. In the latter studies, single fiber proteome profiles were post-analytically classified into their respective types (type I, type IIa, type IIx, type IIb, and hybrid fibers) depending on their myosin isoform abundance.

In the context of focally restricted skeletal muscle alterations, the laser microdissection (LMD) technique provides the possibility to isolate pathogenically relevant tissue or even fiber areas for further proteomic analyses. Using this method, we previously characterized protein aggregates derived from genetically different myofibrillar myopathies [11,29]. Furthermore, this approach led to the identification of proteins that had not previously been described as components of disease-specific protein aggregates. Moreover, we used this approach for the isolation of type I fibers in R349P desmin knock-in mice and delineated a distinct mitochondrial pathology with this desminopathy model [30]. The combination of the immunohistochemical identification of fiber subtypes using MYH isoform-specific antibodies prior to LMD and mass spectrometry (MS) processing allowed us to obtain fiber type-specific proteomes covering up to 1620 proteins as well as to quantify even less abundant proteins. With our method, we were able to analyze the actual proteomic composition of all subfiber types without retrospective annotation.

Our study yielded fiber type-specific proteomic profiles, supporting well-known fiber type characteristics that are commonly reported in the literature. We also provided novel information on fiber type composition and functions. With our protein profiling, we demonstrated that type IIx fibers are not primarily glycolytic, but they do inherit a large number of mitochondrial proteins that are essential for oxidative energetic processes. Our analysis provided us with further evidence that type I and type IIa fibers contain enhanced amounts of proteins known to cause neuromuscular disorders when altered. Our results also suggest that proteins that are essential for accurate cellular maintenance, such as heat shock proteins and the UPS system, have a higher abundance in type I fibers, indicating that there are potential superior rescue mechanisms in this fiber type, as previously reported in [13,31]. With these results, we were able to form a cautious hypothesis on the potential cause of the frequently observed type I fiber predominance. Finally, we developed a hypothesis-free machine learning-based approach, the Random Forest-based Muscle Fiber Peptide Selection Pipeline (FiPSPi), to optimize fiber type assignment by first identifying discriminative peptide panels and then confirming our discriminative peptide panel using a public data set. We are now able to offer our confirmatory R-Script for other researchers, and we plan to standardize our discriminative R-Script to enable the identification of other peptide panels.

## 2. Materials and Methods

### 2.1. Animals

The WT mice used in this study were housed under specific and opportunistic pathogen-free (SOPF) conditions with free access to water and food in a standard environment. Health monitoring was carried out as recommended by the Federation of European Laboratory Animal Science Associations (FELASA). Mice were handled in accordance with the German Animal Welfare Act and the German Regulation for the protection of animals used for experimental purposes or other scientific purposes. All investigations were approved by the responsible governmental animal care and use the office (North Rhine-Westphalia State Agency for Nature, Environment and Consumer Protection (LANUV), Recklinghausen, Germany; reference number 84-02.05.40.14.057). Detailed information on sex, genotype, and age can be found in Table 2.

### 2.2. Preparation of Skeletal Muscle Cryosections, Immunohistochemistry, and Immunofluorescence Staining

Mice were sacrificed, and skeletal muscle specimens were collected and immediately frozen in isopentane precooled in liquid nitrogen. Soleus and tibialis anterior muscles were cryosected into 10 µm thick slices (Cyrostat Microm HM550, Thermo Fisher Scientific, Schwerte, Germany) and placed on a PET membrane frame slide for laser microdissection (LMD). Staining was carried out as described in [32], except that primary antibody incubation was carried out overnight. Antibodies directed against the specific MYH isoforms were purchased from the Developmental Studies Hybridoma Bank (DSHB Iowa City, IA, USA) and used as described in Table 3. PET membrane slides were completely dried prior to LMD.

### 2.3. Laser Microdissection

Laser microdissection (LMD; 6500, Leica Microsystems, Wetzlar, Germany) was carried out as described in [32] except that a total fiber area of 1,000,000 µm^2^ (corresponding to approximately 500 excised fibers) was excised for further analysis with mass spectrometry. Fiber types were excised at 20× magnification. LMD parameters, such as laser intensity, cutting speed, and fluorescence intensity, were adjusted for each PET membrane slide separately to ensure an optimal sample outcome. After the LMD process, samples were covered with 40 µL of formic acid and incubated for 20 min at RT. Sonication of samples for 5 min led to tissue and PET membrane disruption. Samples were stored at −80 °C until further processing.

### 2.4. Lysis of Whole Soleus and Tibialis Anterior Muscles for Spectral Library Generation

Complete murine soleus and tibialis anterior muscles were pulverized in liquid nitrogen, homogenized on ice, and then resuspended in urea buffer (7 M urea, 2 M thiourea, 20 mM Tris base, pH 8.5) as described in [33]. Resuspended samples were sonicated six times for ten seconds with ten seconds rest on ice to support the lysis. The protein concentration was determined by Bradford assay.

### 2.5. In-Solution Tryptic Digestion

In-solution tryptic digestion was performed as described in [30]. In brief, formic acid was completely removed by vacuum vaporization (Vacuum concentrator RCV2-25 CD Plus, Martin Christ Gefriertrocknungsanlagen, Osterode, Germany), and the total digestion volume was set to 50 µL using 50 mM ammonium bicarbonate and 5 mM 1,4-dithiothreitol (DTT) (end concentration). Reduction with DTT was performed for 20 min at 56 °C followed by alkylation for 15 min in the dark at room temperature with 15 mM IAA (end concentration). Tryptic digestion was carried out overnight using 0.1 µg of trypsin per sample, and the reaction was stopped after 16 h by adding 0.5% trifluoroacetic acid (TFA) (end concentration). The sample volume was completely vaporized in a vacuum concentrator, and peptides were solved in 50 µL of 0.1% TFA.

### 2.6. High pH Reversed-Phase Fractionation

Fractionation of the highly complex muscle sample enabled higher proteome coverage and, through that, high protein identification rates in the MS analysis. High pH reversed-phase fractionation of digested muscle samples was performed using the Pierce™ High pH Reversed-Phase Peptide Fractionation Kit (Thermo Fisher Scientific, Bleiswijk, The Netherlands), as recommended in the manual. Briefly, 50 µg of digested muscle lysate was loaded onto the equilibrated spin column, and an increasing organic solvent gradient led to the fractionation of peptides. In total, 8 fractions were collected and used for spectral library generation.

### 2.7. MS Analysis

The mass spectrometry proteomics data have been deposited into the ProteomeXchange Consortium via the PRIDE [34] partner repository with the data set identifier PXD025359.

#### 2.7.1. Data Dependent Acquisition (DDA) for Spectral Library Generation

Nano HPLC analysis was performed on an UltiMate 3000 RSLC nano LC system (Thermo Fisher Scientific, Bremen, Germany), as described in [11]. The HPLC system was online-coupled to the nano ESI source of a QExactive HF mass spectrometer (Thermo Fisher Scientific, Bremen, Germany). In the ESI-MS/MS analysis, full MS spectra were acquired in the range from 350 to 1400 m/z with a resolution of 60,000 at 200 m/z for the detection of precursor ions (AGC target 3 × 10^6^, 80 ms maximum injection time). The m/z values initiating MS/MS were set on a dynamic exclusion list for 30 s, and the ten most intensive ions (charge state +2, +3, +4) were selected for fragmentation. MS/MS fragments were generated by high-energy collision-induced dissociation (HCD). The normalized collision energy (NCE) was either set to a fixed value of 27, or a stepped NCE was applied (25.5, 27, 30). The fragments were analyzed in an Orbitrap analyzer with a 30,000 resolution at 200 m/z (AGC 1 × 10^6^, maximum injection time 120 ms).

#### 2.7.2. Spectral Library Generation

Data-independent acquisition (DIA) regularly uses a spectral library to ensure the correct assignment of fragment ion spectra to their respective precursor ions. To generate an optimal DIA method, a sample-specific library was created using laser microdissected muscle tissue samples as well as whole soleus and tibialis anterior muscles (Spectral library Supplementary Tables S1 and S2). All samples were lysed and partially fractionated by high pH fractionation prior to DDA analysis. Prior to analysis, all samples were labeled by using internal retention time (iRT) peptides (Biognosys, Schlieren, Switzerland), as recommended by the vendor’s protocol. Different gradients, as well as NCE settings, were included in the library to extend the protein identification rates, resulting in a total of 56 DDA runs (for annotated raw files, see Supplementary Table S3). Spectral library generation was carried out using Spectronaut^TM^ Pulsar and the Pulsar search engine (v. 12.0.20491.7.17149, Biognosys, Schlieren, Switzerland). Data were searched against the Uniprot KB *mus musculus* reference proteome set, including iRT peptides (version 2018_06, 53,560 entries) and a contaminant database, resulting in a library size of 28,004 peptides and 5206 proteins. Biognosys factory settings were applied, and trypsin was chosen as the digestion enzyme. Specific modifications were set according to the sample treatment: carbamidomethylation (C) (fixed modification) and oxidation of (M), deamidation (NQ), and carbamidomethylation (N-term) (variable modifications) were included.

#### 2.7.3. Data-Independent Acquisition (DIA) for Differential Proteome Analysis

Prior to DIA, labeling of samples by internal retention time (iRT) calibration was performed for the evaluation of data with Spectronaut^TM^ Pulsar. iRT peptides were prepared as recommended by Biognoysis, and a volume of 1 µL per sample was spiked in the prior analysis. All steps of DIA were again carried out on the QExactive HF using an 8 µL sample volume. In the ESI-MS/MS analysis, full MS spectra were scanned in a range from 350 to 1400 m/z with a resolution of 120,000 at 200 m/z for the detection of precursor ions (AGC target 3 × 10^6^, 20 ms maximum injection time). MS/MS fragments were generated by high-energy collision-induced dissociation (HCD) in which ion dissociation was performed at an NCE of 27%, a fixed first mass of 130.0 m/z, and 24 isolation windows of 45 m/z. The fragments were analyzed in an Orbitrap analyzer with 30,000 resolution at 200 m/z (AGC 1 × 10^6^, maximum injection time 120 ms).

### 2.8. Absolute Protein Concentration Estimation Using aLFQ

After exporting the non-normalized protein and peptide intensities out of Spectronaut Pulsar (Supplementary Tables S4 and S5), locally weighted scatter plot smoothing normalization (LOESS) was applied using the limma R package, version 3.36.5 [35,36]. Calculation of protein quantities was performed using the aLFQ package (version 1.3.5 [37]) within R (version 3.6.1). First, for each protein, an iBAQ value [38] was calculated from the LOESS-normalized peptide intensities (Supplementary Table S6). Absolute protein quantities were obtained using the known total protein concentration of 200 ng per sample. In aLFQ, this is achieved by dividing all iBAQ values from all samples by the total sum of iBAQ values and multiplying this with the specified total protein concentration. We modified the aLFQ workflow and conducted the protein quantification step separately for each sample to ensure there was a total normalized protein intensity of 200 ng in each sample. The modified R-script (aLFQ) is provided in the Supplementary Material. Percentage values were calculated determined by calculating the sum of all protein aLFQ values for each sample separately. The resulting sum value was equal to 100%. Subsequently, single protein percentage values were calculated for each protein and each sample.

### 2.9. Hierarchical Clustering and Subsequent go Term Enrichment and Pathway Enrichment

Non-normalized data obtained from Spectronaut were normalized as described above and used for unsupervised hierarchical clustering with Perseus (v. 1.6.1.3) [39]. For this, the normalized values were log transformed. Prior to clustering, the data set was filtered by setting a minimum percentage of valid values of 50%. Existing missing values were replaced using a normal distribution by imputing with a width of 0.3 and a downshift of 1.8. Groups were averaged using the medians, and data were Z-scored prior to clustering. For unsupervised hierarchical clustering, the Euclidian distance was chosen with an average linkage and no constraint. K-means was enabled, and a value of 300 clusters was chosen with a maximal number of 10 iterations. The resulting clusters were exported and used for subsequent enrichment analysis.

Pathway and Gene annotation enrichment analyses were carried out using DAVID Bioinformatics Resources 6.8 [40,41,42]. For this, the functional annotation tool was used. Uniprot accession of proteins was uploaded. Here, only the first accession for protein group results was used to enable accurate analysis. The Uniprot ID identifier was set, and the list type used was the gene list. As a background, the whole *mus musculus* genome was chosen. The KEGG pathway was used for the determination of enriched pathways in the data set. The term “cellular compartment” was chosen for GO term enrichment studies. Fold enrichment scores, as well as FDR, were used as additional options. Threshold factory settings were maintained. The resulting tables were exported into excel and ranked using p-value corrected values or fold enrichment scores.

### 2.10. Random Forest-Based Muscle Fiber Peptide Selection Pipeline (FiPSPi)

In order to identify a discriminative peptide panel for optimized fiber type assignment by a hypothesis-free machine learning-based method, the Random Forest-based Muscle Fiber Peptide Selection Pipeline (FiPSPi) was implemented as part of this work. FiPSPi was implemented in the Medizinisches Proteom-Center and BioInfra.Prot [43] in R programming language [44] using the R packages randomForest [45] and rpart [46]. Currently, FiPSPi consists of two R scripts, one to identify panels of discriminative peptides and one to confirm them with independent data. The original FiPSPi code is available as supplementary material for this paper, and further developed versions can be downloaded from GitHub (https://github.com/mpc-bioinformatics/FiPSPi, accessed on 11 November 2019).

### 2.11. Validation by Normalized Quantitative Western Blotting Based on Standardized Fluorescent Labeling

Due to the very limited amount of LMD tissue, whole murine soleus and tibialis anterior muscles were used for the Western Blot analysis to validate the findings of the global protein analysis [47,48]. Soleus and tibialis anterior muscles were lysed as described in [33], and 50 µg of protein lysate was separated by 1D-PAGE (polyacrylamide gel electrophoresis) (50 V for 15 min and 180 V for 1 h) using MES running buffer (50 mM MES, 50 mM Tris, 2 mM EDTA). The Quantitative Smart Protein Layers (SPL) Western Blotting system (NH DyeAGNOSTICS GmbH, Halle, Germany) was used for the detection of quantitative differences between selected candidate proteins, and it was used as recommended in the vendors’ protocol. In short, the total protein was pre-labeled with a red fluorescent fluorophore (700 nm detection wavelength), and a 12.5 kDa green fluorescence-labeled standard protein was spiked in (800 nm detection wavelength), enabling error correction of differing sample loadings and data normalization between experiments. The resulting gel was scanned at wavelengths of 700 and 800 nm prior to blotting to determine the total protein and spike in protein quantities and identify inconsistent loss during the blotting procedure. Semi-dry Western blotting was performed at 128 mA for one hour using two different buffer systems (Anode buffer: 300 mM Tris, 100 mM Tricine pH: 8.7, Cathode buffer: 300 mM6-Aminocaproic acid, 30 mM Tris pH: 8.7). Antibodies for desmin (DAKO, Agilent, Frankfurt, Germany 1:100) and myotilin (Santa Cruz, Heidelberg, Germany, 1:200) were used for validation. Incubation was carried out overnight at 8 °C. Blots were washed three times with 1× TBS and incubated with secondary antibody (IRDye 800CW, LI-COR Biosciences, Bad Homburg, Germany, Goat anti-mouse, 1: 10,000) for one hour at room temperature. After washing, Western blots were scanned at wavelengths of 700 and 800 nm using the Odyssey^®^ imaging system (LI-COR Biosciences, Bad Homburg, Germany). Quantification of results was carried out using the vendor’s software (SPL LabImage Software), as described by the vendor’s protocol, resulting in quantitative protein volumes (SPL normalized volume).

## 3. Results

### 3.1. Study Overview and Proteomics Workflow

Recent fiber type classification methods rely on immunolabeling of myosin heavy chain (MYH) isoforms in skeletal muscle tissue specimens. We used this approach for the precise excision of skeletal muscle fiber types out of complex muscle tissue using laser microdissection (LMD) followed by data-independent acquisition (DIA) mass spectrometry (MS) to uncover differences in four murine skeletal muscle fiber type proteomes. For this, 15 mice were euthanized, and their soleus and tibialis anterior muscles were excised, cryosected, and stained for the collection of type I, type IIa, type IIb, and type IIx fibers. MYH isoform-specific antibodies enabled the distinction between fiber types and their selective isolation (Supplementary Figure S1). Using an MS-based approach, whole muscle tissue and LMD muscle tissue were analyzed, resulting in the identification of 5206 muscle-specific proteins. This in-depth proteome served as a reference for the subsequent fiber type characterization. The analysis of excised fiber types (total number of fiber type samples *n* = 56) resulted in the identification of over 1600 (1037 quantified) different fiber-specific proteins using a minimal amount of sample (approximately 150 ng of peptide extract). The fiber-specific proteome analysis revealed 889 proteins in type I, 875 proteins in type IIa, 738 proteins in type IIb, and 800 proteins in type IIx fibers.

### 3.2. Fiber Types Are Distinguishable at the Proteomic Level

**Figure 1:**
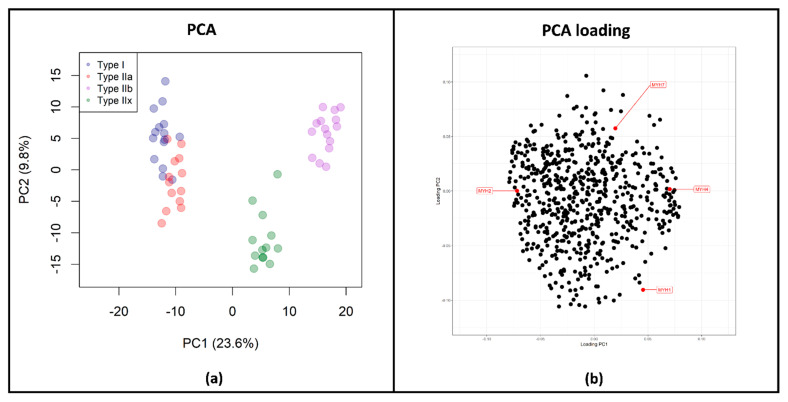
Differentiation among the four fiber types. (**a**) Principal component analysis (PCA) of fiber types (*n* = 56). Significant separation of type IIb (purple) and type IIx fibers (green) can be observed in principle component (PC2), while type I (blue) and type IIa fibers (red) formed a combined, but significantly separated, cluster from type IIx and type IIb fibers in PC2 with moderate segregation into type I and type IIa fibers. (**b**) PCA loadings, visualizing the proteins separating the four groups. The four fiber type characteristic myosin heavy chain (MYH) isoforms are indicated in red and were identified as major separators.

In the first step, we assessed whether the different fiber subtypes could be generally distinguished based on their protein profiles. For this, sample correlation was carried out by principal component analysis (PCA) for each fiber type sample (Figure 1a). A significant separation of type IIb and type IIx fibers was observed, while type I and type IIa fibers formed a combined, but significantly separated, cluster from type IIx and type IIb fibers with moderate segregation into type I and type IIa fibers. From these results, we concluded that type I and type IIa fibers have similar protein profiles. Type IIb and type IIx fibers, on the other hand, harbor unique proteomic profiles that are clearly distinguishable from those of all other fiber types. In order to identify the strongest separating factors, PCA loadings were examined (Figure 1 and Supplementary Table S7). We confirmed that the four fiber type characteristic MYH isoforms were the major separators (Figure 1b), supporting their fiber type specificity. However, well-known slow and fast fiber type marker proteins, such as specific isoforms of troponin (troponin I, troponin T) [49], myomesin (myomesin-1, myomesin-2), and myozenin (myozenin-1, myozenin-2) [27,50] were found to be essential for fiber type separation. Moreover, a fiber type-specific shift in the distribution of heat shock and proteasomal proteins was observed (segregation toward type I and type IIa fibers), suggesting there is fiber type-specific protein expression for a variety of functional groups (for further details, see Supplementary Table S8).

The current gold standard for fiber type discrimination is based on the detection of MYH isoforms with the following assignments: MYH7 for type I, MYH2 for type IIa, MYH4 for type IIb, and MYH1 for type IIx fibers. Thus, we decided to examine the quantity distribution for each MYH isoform in all fiber types using representative peptides with high-intensity and suitable detectability (Table 4, Supplementary Table S9).

Overall, for each fiber type, the characteristic MYH isoform was detected with the highest abundance but was not the only isoform present. MYH4 showed the most distinct pattern in type IIb fibers with neglectable levels of other isoforms. Type I fibers instead displayed high intensities not only for MYH7 but also for MYH2. At this stage, our results show that the expected MYH isoforms are all present in the respective fibers, but the presence of other forms is also indicated. We further focused our attention on other known marker proteins, especially sarcomeric proteins for slow and fast fibers, which have been verified in several studies [27,49,51,52,53] (Table 5, Supplementary Table S8). Type I and type IIb fibers could unequivocally be identified by having a high abundance of either slow (type I) or fast (type IIb) sarcomeric protein markers, such as different isoforms of myomesin and myozenin (Table 4). Notably, the traditionally classified fast type IIa fibers showed an enhanced concentration of slow fiber type protein markers, such as alpha-actinin-2, myozenin-2, and troponin C. Type IIx fibers could not be unambiguously classified as either slow or fast, as they displayed a high expression of marker proteins that are indicative of fast but also slow fibers.

**Figure 2:**
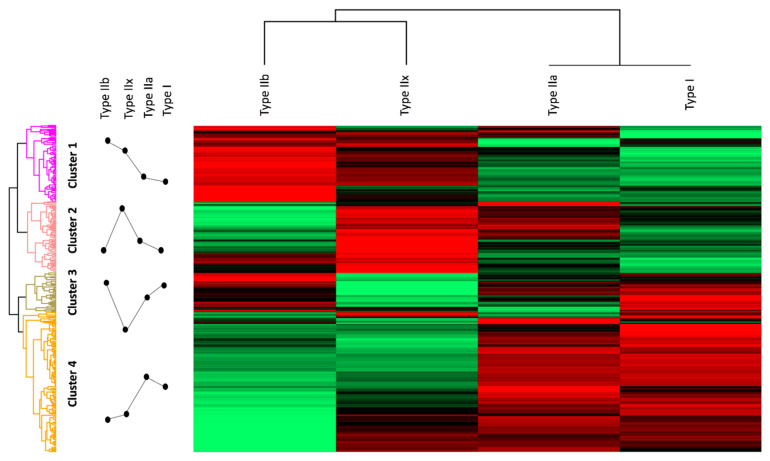
Hierarchical clustering: high protein intensities are shown in red, and low protein intensities are shown in green. The profile plots indicate trends of protein expression intensity in the different fiber types. Cluster 1 displays high intensities in type IIx and type IIb fibers. Proteins of cluster 1 were annotated to glycolysis, calcium signaling, and the sarcoplasmic reticulum. High intensities in cluster 2 were annotated to type IIa and type IIx fibers and could be associated with mitochondrial processes such as the tricarboxylic acid (TCA) cycle the oxidative phosphorylation (OXPHOS) pathway and the mitochondrial respiratory chain complexes. Cluster 3 shows high-intensity values in type I, type IIa, and type IIb and is composed of ribosomal and proteasomal proteins. Cluster 4 can be annotated to structural components of the sarcomere as well as mitochondrial processes, such as the OXPHOS pathway, the TCA cycle, and the respiratory chain but also has a unique annotation to fatty acid beta oxidation and high intensities in GO terms and pathways associated with structural components of the sarcomere and the cytoskeleton.

In the next step, we performed a cluster analysis, resulting in 4 distinct protein expression clusters, (see Figure 2) and examined them by GO (gene ontology) term and pathway enrichment analyses (Supplementary Table S10, Supplementary Figures S2–S9). The formation of clusters is based on combining proteins with similar expression profiles into a common cluster. Each protein is assigned only once, resulting in unique clusters. This can provide information about the individual protein profiles of individual fiber types but also about similarities between them. As described in the literature, fast fiber types are highly glycolytic, as they need a fast but nonpersistent supply of energy. Accordingly, members of cluster 1, which were mainly annotated as enzymes involved in glycolysis, sarcomere, and cytoskeleton-associated proteins and proteins involved in calcium release and storage, were found to be associated with type IIb and type IIx fibers. Notably, cluster 1 was found to be the only cluster displaying GO terms associated with the sarcoplasmic reticulum, the central storage area for calcium within the muscle (Supplementary Table S10 and Supplementary Figures S2 and S6), underlying the unique twitching ability of fast type IIx and type IIb fibers. Members of cluster 2 (137 proteins) were found to be mainly associated with type IIa and type IIx fibers. This cluster primarily comprised proteins involved in oxidative processes. The GO term enrichment analysis revealed a high level of enrichment of mitochondria-associated terms, such as the “respiratory chain” and the “mitochondrial proton-transporting ATP synthase complex”, indicating that the oxidative energy supply is of high importance in both fiber types (Supplementary Table S10, Supplementary Figures S3 and S7). In cluster 3 (77 proteins), proteins involved in protein homeostasis (e.g., ribosomes and proteasomes) and proteins associated with the Z-disk were found (Supplementary Table S10, Supplementary Figures S4 and S8). Here, type I, type IIa, and type IIb fibers showed comparably high abundances, while type IIx fibers displayed lower values. Type I fibers known to contain a large number of mitochondria displayed a large number of proteins involved in mitochondrial processes assigned to cluster 4, such as essential energetic processes like the respiratory chain and fatty acid beta oxidation (cluster 4, 271 protein members (Supplementary Table S10, Supplementary Figures S5 and S9)). Type IIa fibers showed comparable expression patterns in this cluster, which is in accordance with their high oxidative energetic potential. Notably, cluster 4 contained a high number of GO terms and pathway annotations associated with structural elements of the sarcomeres, cytoskeleton, and extracellular matrix. Among others, the GO terms “dystroglycan complex” and “sarcoglycan complex” showed fold enrichment scores of more than 45-fold. Additionally, the terms “M-band”, “I-band”, “A-band”, and “Z-disk” were found to be common with fold enrichments of more than 10-fold.

Although the GO term and pathway annotations of all clusters showed similarities either with respect to oxidative-energy-providing processes and/or sarcomere structure, proteins within each cluster differed, as all proteins were assigned to a cluster only once. Nevertheless, the GO term and pathway analysis using DAVID resulted in multiple annotations for several proteins. Therefore, we decided to manually annotate each protein into a single group based on pathway or localization, and we calculated their percentage values with respect to cluster size (Table 6 and Supplementary Table S10a). Our manual annotation of cluster members revealed that cluster 2 exhibited the highest number of proteins associated with respiratory chain complexes (53 proteins in cluster 2 (37.23%), 4 proteins in cluster 3 (5.19%), and 32 proteins in cluster 4 (7.38%)). Cluster 4, instead, displayed a remarkably high number of proteins associated with fatty acid beta oxidation (14 proteins, (5.17%)). Although the content of proteins associated with structural components of the sarcomere and the cytoskeleton were similar in cluster 1 and cluster 4, our manual annotation revealed that cluster 1 mainly exhibited proteins associated with fast-twitch muscles, such as fast troponin isoforms and myosin-4. Cluster 4, instead, displayed enhanced intensities for slow fiber type markers and components of the extracellular matrix (Supplementary Table S10a).

In addition to this, previously unknown characteristics were revealed, such as increased mitochondrial protein content in type IIx fibers. In comparison with type IIb fibers, all other fiber types showed a significantly greater abundance of proteins that are essential for the oxidative supply of ATP, a feature that the literature mainly attributes to slow type I fibers and, to a lesser extent, to fast type IIa fibers. According to our data, type IIx fibers behave more as intermediate fibers: they contain the highest numbers of both oxidative and glycolytic enzymes (Supplementary Table S8). Type IIb fibers, on the other hand, showed a high percentage of glycolytic enzymes and proteins involved in calcium influx and efflux, which is typical for fast fibers. Compared with all other groups, type IIb fibers exhibited the clearest fast fiber phenotype. This proteomic classification indicates a high level of similarity between slow-twitch type I and fast-twitch type IIa fibers and provides novel information on the intermediate nature of type IIx fibers (see Table 7).

### 3.3. Definition of a Discriminative Peptide Panel for Optimized Fiber Type Assignment

**Figure 3:**
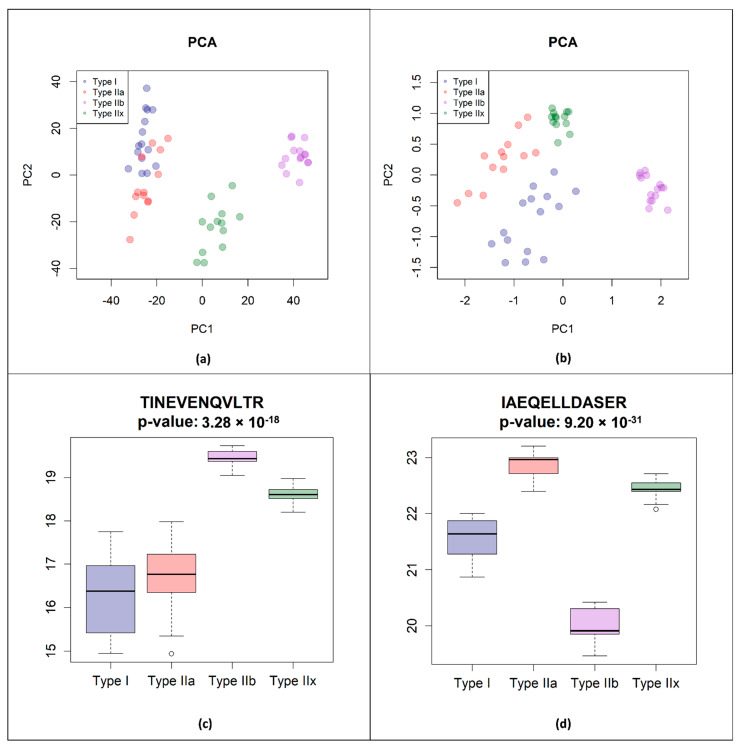
(**a**) Principal component analysis (PCA) for all fiber types (*n* = 56) based on our complete proteomic data. Clear discrimination of type IIb and T2X fibers is possible, whereas type I and type IIa fibers cluster together. (**b**) PCA based on our alpha-actinin-3 and myosin-1 peptide panel. Discrimination of type I and type IIa fibers is especially enhanced. (**c**,**d**) Boxplots of the intensity value distributions on a log2-scale of the four fiber types, type I, type IIa, type IIb, and type IIx, for alpha-actinin-3 peptide (**c**) and the myosin-1 peptide (**d**), which were used in our panel. The alpha-actinin-3 peptide TINEVENQVLTR differentiates well between the type IIa, type IIb, and type IIx fibers, and the myosin-1-peptide IAEQELLDASER differentiates well between type I and all other fiber types, especially type IIa. Thus, only these two peptides are needed to differentiate all four fiber types.

To further optimize the clear allocation of the four different fiber types, we used a hypothesis-free machine learning-based feature selection and classification approach with random forests [54] (i.e., random forest-based recursive feature elimination [43,55]) to clearly define the discriminatory protein/peptide panel. To do so, we established the Random Forest-based Muscle Fiber Peptide Selection Pipeline (FiPSPi) script to identify peptides that could differentiate between the four fiber types based on our data set. The best discrimination panel consisted of one alpha-actinin-3 (TINEVENQVLTR) and one myosin-1 (IAEQELLDASER) peptide, two well-known fiber type-specific markers for fast fiber types. With this panel, an average classification accuracy value of 99.94% was obtained as well as improved fiber type clustering, giving clear separation of type I and type IIa fibers (see Figure 3b). Moreover, the alpha-actinin-3 peptide was found to differentiate well between type IIa, type IIb, and type IIx fibers, and myosin-1 peptide differentiates well between type I and all other fiber types, especially type IIa (Figure 3c,d).

To further validate the discriminatory potential of these two peptides, we developed a second confirmatory script for peptide validation. To validate our discriminative peptides, an independent public data set, in which single muscle fibers were mechanically dissected and retrospectively annotated, was used [12]. Detailed information about the criteria applied to the public data set and the subsequent samples taken for validation can be found in Appendix A Table A1 and Supplementary Table S11. As the fiber type isolation technique and mass spectrometric methods used differed from our approach, we expected significantly decreased classification results for this data set, where a classification accuracy of about 75–80% would be sufficient to validate the panel. However, an average classification accuracy of 87.2% was achieved using the machine learning-based classification approach described in this study. Here, the alpha-actinin-3 peptide was able to distinguish between type I and the other fiber types, and the myosin-1 peptide could differentiate between type IIx and all other fiber types (Supplementary Figure S11a,b). To make the panel more intuitively applicable for the reader, we further constructed a decision tree for both data sets (Supplementary Figures S10 and S11c), which uses optimized threshold values between the fiber type distributions of the two peptides, which are indicated by the well-separable boxplots presented in Figures S10c,d and S11a,b as classification rules. As can be seen in Supplementary Figure S10, the decision tree-based approach works well for our data set since no ambiguities can be observed in the terminal nodes. Our classification rules could be generalized to other data sets, and this may provide a valuable tool for further studies and clinical applications. To allow this, we commented on the associated R code and made it available in the Supplementary Materials (FiPSPi.zip), as well as in a GitHub repository (https://github.com/mpc-bioinformatics/FiPSPi, accessed on 11 November 2019), in which we aim to generalize the code for other fiber type-based proteomic data sets and thereby enable the further identification of discriminative peptides.

### 3.4. Higher Expression Levels of Muscular Disease Associated Proteins in Type I and Type IIa Fibers

**Figure 4:**
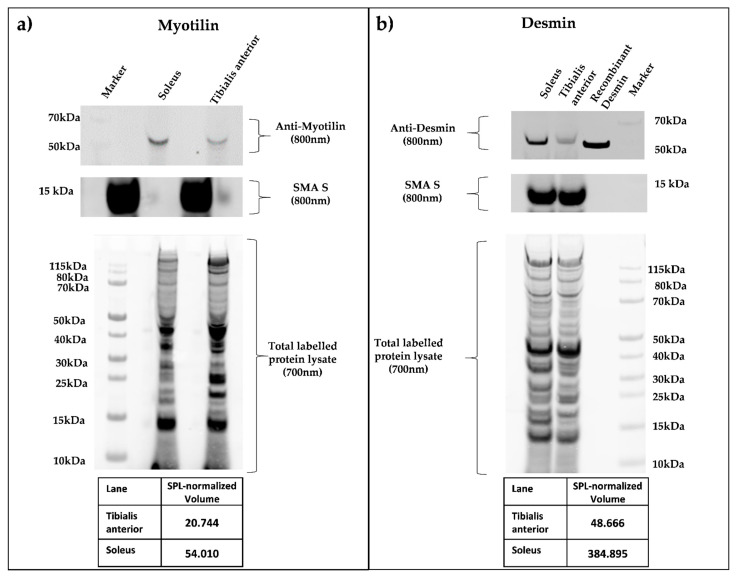
(**a**,**b**) Quantitative Smart Protein Layers (SPL) Western blot analysis of whole murine soleus and tibialis anterior muscle. SPL Western blotting system (NH DyeAGNOSTICS GmbH) was used for the detection of quantitative differences between selected candidate proteins. Total protein was pre-labeled with a red fluorescent fluorophore (700 nm), and a 12.5 kDa green fluorescence-labeled standard protein (SMA S, 800 nm) was spiked in, enabling error correction of differing sample loading and data normalization between experiments. Primary antibodies against myotilin (**a**), myotilin (E-10), sc-393957), and desmin (**b**), DAKO, M0760 Clone D33) were visualized by infrared secondary antibodies (IRDye goat anti-mouse 800 nm). SPL system analysis resulted in quantitative protein volumes (SPL normalized volume), determining a higher concentration in the soleus muscle for both proteins.

Skeletal muscle fibers react differently to changes in life, for instance, aging and exercise, but also in the context of neuromuscular disorders. In order to assess the abundance of various myopathy-related proteins in the four fiber types, we investigated the expression patterns of dystrophin and several myofibrillar myopathy-associated proteins in more detail. The latter group comprised desmin [56], αB-Crystallin [57], myotilin [57,58], Z-band alternatively spliced PDZ motif-containing protein (ZASP) [59,60], filamin-C [61], BAG family molecular chaperone regulator 3 [62], the four-and-a-half-LIM protein 1 [63], plectin, alpha actin 1, heat shock protein B8, and DnaJ heat shock protein family member B6 [64]. Our detailed analyses of the profiles of these proteins revealed that dystrophin displayed enhanced expression levels in type I and type IIa fibers. Additionally, proteins involved in forming the dystrophin-associated glycoprotein complex, such as dystrophin, beta-sarcoglycan, delta-sarcoglycan, and gamma-sarcoglycan, were all found to be over-represented in slow type I fibers when compared with fast type IIb fibers (Supplementary Table S8). With respect to myofibrillar myopathy-associated proteins, alpha-crystallin B, desmin, myotilin, BAG family molecular chaperone regulator 3, and heat shock protein beta-8 showed enhanced expression in type I and type IIa fibers. Filamin C and plectin showed comparable expression levels for type I, type IIa, and type IIx fibers (Table 8). Alpha actin instead displayed the highest level of expression in type I fibers, and the four-and-a-half-LIM protein 1, as well as the Z-band, alternatively spliced PDZ motif-containing protein, could not be identified in the data set. We further verified the MS results for desmin and myotilin by a quantitative Western Blot approach (Figure 4) that compared the soleus (solely composed of type I and type IIa fibers) with the tibialis anterior muscle (mainly composed of type IIb and type IIx fibers). In accordance with our proteomic results, myotilin was found to have 3.7 fold higher expression in the soleus muscle, and desmin even showed enrichment of eight-fold in the soleus muscles of healthy WT mice.

In addition, sarcomere-damage-associated components showed increased expression in type I and type IIa fibers. Among these is Xin-actin-binding-repeat-containing protein 2 (Supplementary Table S8). Xin-actin-binding-repeat-containing protein 2 is not only known to selectively stabilize filamin-C [65] but has also recently been identified as a marker of muscular damage [66]. Additionally, a large number of subunits of the 26S proteasome were found to be over-represented in type I and type IIa fibers (Supplementary Table S8). These subunits are essential for the degradation of sarcomeric proteins, as the ubiquitin-proteasome-dependent proteolytic pathway is mainly responsible for the breakdown of myofibrillar proteins [67].

In summary, our analysis broadens our insight into the specific protein composition of muscle fiber types. Our analysis highlights that type I and type IIa fibers, apart from type IIx and type IIb fibers, share similar protein profiles. Further, type IIx fibers, which are traditionally classified as highly glycolytic, showed enhanced expression of proteins associated with an oxidative energy supply. Moreover, our study revealed that type I and type IIa contain higher concentrations of myopathy-related proteins. Additionally, a variety of proteins involved in protein degradation and protein folding were found to be more highly expressed in these two fiber types.

## 4. Discussion

In this study, we set out to characterize the proteomic compositions of different subfibers in the skeletal muscle tissue of healthy mice. Our applied analysis workflow has the following advantages: (i) the protocol requires very little sample material, which makes it highly applicable for small samples, such as those obtained with skeletal muscle biopsies; (ii) our method is not dependent on postanalytical assignment of fiber types and enables ad priori distinction of the four fiber types by immunohistochemical staining and selective enrichment by laser microdissection. This strategy serves as a powerful tool that allows in-depth proteome analysis of fiber types, giving a more distinct view of metabolic differences and susceptibility to pathophysiological processes in different types of skeletal muscle fiber. Type IIa and type IIx fibers showed less distinct MYH isoform profiles, leading us to the hypothesis that these two types of fibers may take on variable functions and tasks according to their composition and function. Although both fiber types are referred to as fast, the literature states that type IIa fibers mainly sustain their energetic needs using oxidative pathways and, therefore, by containing high numbers of mitochondria, whereas type IIx fibers rely on glycolysis to produce ATP rapidly [5,10,68]. Our further analysis identified a more versatile function of type IIx fibers in the body. With our technique, we proved that type IIx fibers inherit both oxidative and glycolytic properties while exhibiting solely fast fiber type marker proteins. Although the adaptive behavior of type IIx fibers and their high content of proteins essential for the oxidative energy supply is rarely documented in the literature, a similar phenomenon has already been observed in other mammalian species, such as springbok and deer. In these species, the oxidative capacity of type IIx fibers could prevent early fatigue of muscles after sprinting [69]. Therefore, the observed dual nature of type IIx fibers might have originated from adaptive muscle requirements, stressing the versatile function of type IIx fibers. Although type IIx fibers were shown to have a dual nature, differences between type IIx, type I, and type IIa fibers in terms of their oxidative energy supply became clear during the course of our analysis. In contrast to type IIx fibers, type I and type IIa fibers displayed increased intensities in proteins involved in fatty acid beta oxidation, confirming that type I and type IIa fibers have a greater ability to provide a long-lasting supply of ATP than other fiber types by breaking down fatty acids. Thus, our results allow the conclusion that the traditional and/or immunohistochemical classification based on ATPase staining and/or MYH isoform expression reflects only some of the molecular characteristics of the different fiber types. Based on our proteomic data, the current classification of fiber types can further be refined by our new insights into the abundances of proteins involved in the metabolic processes of muscle fibers.

Furthermore, we aimed to develop an unbiased approach to reclassify our fiber types. Our machine-learning strategy FiPSPi that was developed in-house allowed us to optimize fiber type assignment, resulting in the selection of a discriminative two-peptide panel. Using this tool, we are able to determine every fiber type at the peptide level. FiPSPi further showed the ability to identify type I, type IIx, and TB fibers correctly in an independent publicly available proteomic single fiber type data set that focused on a different approach of single fiber dissection and annotation. Unfortunately, the group of type IIa fibers could not be included in the validation study because only three samples remained after the application of our stringent criteria (for further information, see Appendix A), which was not sufficient for subsequent statistical testing. However, for type I, type IIx, and type IIb fibers, high classification accuracy was reached, validating our identified discriminative peptide panel. We have deposited the complete FiPSPi code consisting of two R Scripts with a detailed description in the Supplementary Material and in a GitHub repository, which can be reached via https://github.com/mpc-bioinformatics/FiPSPi, accessed on 11 November 2019. The first script is used to identify suitable panels of discriminative peptides between fiber types and is currently built on our data set. The second script is used to validate the discrimination of fiber types by previously identified peptides based on independent proteomic data sets and can already be used by other researchers. Furthermore, we plan to generalize the first script to enable other researchers to analyze their proteomic fiber type-based data sets and thereby potentially identify further discriminative peptides.

We further aimed to investigate our proteomic fiber type data with respect to currently unknown unique fiber type features, as the literature regularly indicates the unique properties of skeletal muscle fiber types and their high adaptability to lifestyle changes [19,28,70,71,72,73,74] and their differential levels of susceptibility in muscular disorders [24,26,75,76].

Strikingly, proteins are known to be involved or mutated in neuromuscular disorders such as myofibrillar myopathies (MFM) type I, type IIa [23,24,60,62,64,77,78], or Duchenne muscular dystrophy (DMD) [79] show a high level of enrichment in type I and type IIa fibers. In our study, we identify five MFM-causing proteins as being enriched in type I fibers (desmin, myotilin, filamin-C, BAG family molecular chaperone regulator 3, and heat shock protein beta-8). This observation seems somehow counterintuitive, as a higher abundance of disease-relevant proteins in type I fibers leads to the conclusion that these fiber types should be potentially affected more severely. Additionally, type I and type IIa fibers likewise have higher mitochondrial contents. Mitochondrial pathology has already been identified for a large number of neuromuscular diseases, which are characterized by extensive mitochondrial abnormalities underlying their potential higher vulnerability during disease progression [30,80]. Indeed, the reason for the frequently observed phenomenon of type I fiber predominance still remains largely unexplained, and studies focusing on this phenomenon have largely concluded that there is selective wasting of fast T2 fibers [26,81].

Nevertheless, our further in-depth proteomic profiling of fibers also revealed an enrichment of proteins essential for the proper folding of sarcomeric components as well as their degradation in type I and type IIa fibers. This leads us to the vague hypothesis that type I and type IIa fibers might inherit superior compensation or advanced rescue mechanisms, preventing the accumulation of misfolded proteins present in several neuromuscular diseases. Indeed, at this level, we cannot say with certainty why type I fiber predominance regularly occurs. Further research is needed, e.g., combining omics techniques and physiological/functional studies, to understand why the phenomenon of type I fiber predominance is observed in numerous diseases.

Finally, it should be noted that our approach also works optimally for clinical samples, as it requires only minimal amounts of tissue and protein and may be further optimized by establishing label-free-based detection of fiber types by coherent anti-Stokes Raman scattering as described in [82]. Needle biopsies of muscles are one of the most common procedures used to study the histological effects of lifestyle changes on skeletal muscles. Deciphering the proteomic composition has emerged as a valid tool to identify the molecular mechanisms involved in skeletal muscles during life progression, and the information gained could be used in clinical applications [11,32]. Our approach may enhance human fiber type analysis by revealing distinct fiber type behaviors.

## Figures and Tables

**Table 1:** Detailed information on myosin isoforms (MYH): accession (Uniprot), protein name, protein name abbreviation, gene name, and corresponding fiber type.

Accession (Uniprot)	Protein Name	Protein Name Abbreviation	Gene Name	Corresponding Fiber Type
Q91Z83	Myosin-7	MYH7	*Myh7*	Type I
G3UW82	Myosin, heavy polypeptide 2, skeletal muscle, adult *	MYH2	*Myh2*	Type IIa
Q5SX39	Myosin-4	MYH4	*Myh4*	Type IIb
Q5SX40	Myosin-1	MYH1	*Myh1*	Type IIx

* further referred to as myosin-2.

**Table 2:** Sample information. Mouse identifier (ID), sex, genotype, and age in months and days for each mouse used in the present study are displayed.

Mouse ID	Sex	Genotype	Age in Months	Age in Days
1	f	wt	21	657
2	f	wt	21	657
3	m	wt	15	466
4	m	wt	14	448
5	m	wt	14	448
6	m	wt	14	448
7	m	wt	13	401
8	m	wt	11	361
9	m	wt	11	361
10	m	wt	11	361
11	m	wt	3	95
12	m	wt	3	95
13	m	wt	3	96
14	f	wt	3	96
15	f	wt	3	96

**Table 3:** Primary antibodies (product numbers Developmental Studies Hybridoma Bank (DSHB), Iowa) and dilutions used for the detection of fiber types (type I, type IIa, type IIb, and type IIx) using the fiber type-specific myosin (MYH) isoform. Secondary antibodies and dilutions necessary for the detection of primary antibody signals.

Primary Antibody/Dilution	Secondary Antibody/Dilution	Fiber Type	MYH Isoform
BA-F8/1:20	TexasRed/1:60	Type I	MYH7
SC-71/1:20	Cy2/1:200	Type IIa	MYH2
BF-F3/1:20	AlexaFluor594/1:500	Type IIb	MYH4
6H1/1:15	AlexaFluor594/1:500	Type IIx	MYH1

**Table 4:** Summed intensities (Sum) of unique peptides (proteotypicity = TRUE) detected for each myosin heavy chain (MYH) isoform that is indicative of fiber types (type I, type IIa, type IIb, and type IIx).

**Protein**	**Peptide Sequence**	**Proteotypicity**	**Sum Type I (10 × 10^6^)**	**Sum Type IIa (10 × 10^6^)**	**Sum Type IIb (10 × 10^6^)**	**Sum Type IIx (10 × 10^6^)**
MYH7	DTQIQLDDAVR	TRUE	226.57	45.22	0.00	0.00
MYH7	IEDEQALGSQLQK	TRUE	162.65	34.33	0.05	0.19
MYH7	ANDDLKENIAIVER	TRUE	137.41	27.70	0.01	0.01
MYH7	GQNVQQVSYAIGALAK	TRUE	80.46	15.95	0.04	0.03
MYH7	SLQSLLK	TRUE	74.67	15.45	0.02	0.03
**Protein**	**Peptide Sequence**	**Proteotypicity**	**Sum Type I (10 × 10^6^)**	**Sum Type IIa (10 × 10^6^)**	**Sum Type IIb (10 × 10^6^)**	**Sum Type IIx (10 × 10^6^)**
MYH2	LINDLTTQR	TRUE	95.23	216.18	8.32	14.98
MYH2	AAYLQGLNSADLLK	TRUE	77.60	179.82	8.17	11.76
MYH2	VLNASAIPEGQYIDSK	TRUE	74.62	173.66	6.23	11.20
MYH2	GQTVEQVTNAVGALAK	TRUE	39.80	106.67	3.76	7.45
MYH2	IEDEQAIGIQLQK	TRUE	25.58	71.06	2.53	5.65
**Protein**	**Peptide Sequence**	**Proteotypicity**	**Sum Type I (10 × 10^6^)**	**Sum Type IIa (10 × 10^6^)**	**Sum Type IIb (10 × 10^6^)**	**Sum Type IIx (10 × 10^6^)**
MYH4	VAEQELLDASER	TRUE	3.78	3.82	221.71	49.27
MYH4	LINELSTQK	TRUE	4.17	4.55	175.11	37.76
MYH4	AAYLTSLNSADLLK	TRUE	1.87	2.98	129.68	24.67
MYH4	LQDAEEHVEAVNSK	TRUE	1.42	1.34	75.10	17.27
MYH4	RVAEQELLDASER	TRUE	1.52	2.48	74.18	19.12
**Protein**	**Peptide Sequence**	**Proteotypicity**	**Sum Type I (10 × 10^6^)**	**Sum Type IIa (10 × 10^6^)**	**Sum Type IIb (10 × 10^6^)**	**Sum Type IIx (10 × 10^6^)**
MYH1	AAYLQNLNSADLLK	TRUE	14.02	20.54	25.48	122.96
MYH1	SALAHALQSSR	TRUE	14.19	20.28	17.76	97.12
MYH1	DSLVSQLSR	TRUE	8.37	11.42	13.79	49.63
MYH1	NAYEESLDHLETLKR	TRUE	4.60	6.71	8.68	41.85
MYH1	QLDEKDSLVSQLSR	TRUE	4.59	6.82	8.77	41.53

**Table 5:** Percentage values of sarcomeric protein markers and their indication for either slow or fast fibers in all four skeletal muscle fiber types (type I, type IIa, type IIb, and type IIx).

Protein Names	Indicative For	% Type I	% Type IIa	% Type IIb	% Type IIx
Alpha-actinin-2	Slow	2.60	1.89	0.39	1.15
Alpha-actinin-3	Fast	0.10	0.10	0.93	0.63
Myomesin 2	Fast	0.05	0.11	0.36	0.34
Myomesin-1	Slow	0.04	0.02	0.00	0.00
Myosin-binding protein C. slow type	Slow	0.09	0.09	0.04	0.06
Myosin-binding protein C. fast type	Fast	0.08	0.11	0.64	0.33
Myozenin-1	Fast	0.10	0.19	0.30	0.31
Myozenin-2	Slow	0.20	0.14	0.01	0.02
Troponin C. slow skeletal and cardiac muscles (TN-C)	Slow	0.45	0.17	0.00	0.00
Troponin I. fast skeletal muscle (troponin I. fast-twitch isoform)	Fast	0.06	0.12	0.23	0.26

**Table 6:** Percentages of manually annotated proteins into 13 groups (Sarcomere/Cytoskeleton, Respiratory chain, Tricarboxylic acid cycle, Glycolysis, Glycogenolysis, Mitochondria, Proteasome, Heat shock, Nucleus, Fatty acid beta oxidation, Calcium regulation/transport, Ribosome, Others) for each cluster.

Annotation	Cluster 1 (%)	Cluster 2 (%)	Cluster 3 (%)	Cluster 4 (%)
Sarcomere/Cytoskeleton	18.24	5.11	14.29	19.56
Respiratory chain	3.38	37.23	5.19	7.38
Tricarboxylic acid cycle	0.68	7.30	1.30	1.48
Glycolysis	4.73	0.00	0.00	0.74
Glycogenolysis	5.41	0.73	0.00	0.00
Mitochondria	0.00	9.49	1.30	5.90
Proteasome	0.00	0.00	9.09	4.43
Heat shock	0.00	0.00	2.60	3.69
Nucleus	0.68	0.00	2.60	3.32
Fatty acid beta oxidation	2.03	0.73	1.30	5.17
Calcium regulation/transport	8.78	0.73	0.00	1.85
Ribosome	8.78	4.38	28.57	4.43
Other	47.30	34.31	33.77	42.07

**Table 7:** Classification of fiber types according to the traditional classification based on ATPase staining, the oxidative phosphorylation capacity, the immunohistochemical classification based on myosin heavy chain (MYH) isoforms, and the classification based on the proteomic data.

Fiber Type	Traditional Classification (Based on ATPase Staining)	Oxidative Phosphorylation Capacity	Immunohistochemical Classification via MYH Isoform	Proteomic Classification
Type I	Slow	Very high	MYH7	Highly oxidative
Type IIa	Fast	High	MYH2	Highly oxidative
Type IIb	Fast	Very low	MYH4	Highly glycolytic
Type IIx	Fast	Low	MYH1	Oxidative and glycolytic

**Table 8:** Mass spectrometry-based calculated percentages of disease-related proteins and their distribution (in%) in skeletal muscle fiber types (type I, type IIa, type IIb, and type IIx). All proteins do show a higher abundance in type I and type IIa fibers.

Protein Names	% Type I	% Type IIa	% Type IIb	% Type IIx
Actin. alpha skeletal muscle	30.49	24.23	20.14	21.98
Alpha-crystallin B chain	0.06	0.06	0.01	0.02
BAG family molecular chaperone regulator 3	0.01	0.01	0.00	0.01
Desmin	0.37	0.40	0.14	0.20
Dystrophin	0.08	0.07	0.02	0.02
Filamin-C	0.12	0.15	0.08	0.13
Heat shock protein beta-8	0.01	0.01	0.00	0.00
Myotilin	0.38	0.41	0.13	0.25

## Data Availability

The mass spectrometry proteomics data have been deposited into the ProteomeXchange Consortium via the PRIDE [34] partner repository with the data set identifier PXD025359.

## Supplementary Materials

The following are available online at https://www.mdpi.com/article/10.3390/proteomes9020028/s1: Figure S1: MYH Isoform Staining. Figure S2: Bubbleplot_GO Term CC Cluster 1. Figure S3: Bubbleplot_GO Term CC Cluster 2. Figure S4: Bubbleplot_GO Term CC Cluster 3. Figure S5: Bubbleplot_GO Term CC Cluster 4. Figure S6: Bubbleplot_Pathway Cluster 1. Figure S7: Bubbleplot_Pathway Cluster 2. Figure S8: Bubbleplot_Pathway Cluster 3. Figure S9: Bubbleplot_Pathway Cluster 4. Figure S10: Decision Tree. Figure S11: Peptide_Panel_Decision_Tree_Public_Data_Set. Table S1: Spectral library information. Table S2: Spectral library. Table S3: Study annotation. Table S4: Protein_Report_Fiber_types_Spectronaut_Pulsar_no_norm. Table S5: Peptide_Report_Fiber_types_Spectronaut_Pulsar_no_norm. Table S6: Peptide_loess_originalscale_for_aLFQ. Table S7: PCA_loading. Table S8: aLFQ. Table S9: Peptides_MYH_isoform_distribution. Table S10: GO_Term_Pathway_analysis_DAVID. Table S10a: Functional_annotation_Clusters. Table S11: Public Data set_Samples. FiPSPi.zip. Rcode aLFQ.

## Appendix A

### FiPSPi Validation of Public Data Set Exclusion Criteria

As an independent validation data set for the identified two-peptide panel, search files from Murgia et al. [12] were downloaded from the PRIDE repository (PXD001641). The unnormalized peptide intensities were normalized using the same method as that described in Section 2.8 of the Materials and Methods Section in the main manuscript (i.e., LOESS normalization). Due to the metadata available as supplementary information for the original paper by Murgia et al., not all samples from the original data set were appropriate for panel confirmation within this work. Consequently, exclusion criteria were formulated to exclude particular samples from the validation data set that were not appropriate and/or useful for the confirmation of the identified two-peptide panel described in the main manuscript.

The following exclusion criteria were applied:

- Samples without a predominant MYH isoform and/or without an unambiguous fiber type in the column “Fiber type, renamed” in the Supplementary Material for Murgia et al. (Supplementary Table S1, file *embr201439757-sup-0002-supp_tables1.xlsx*) were excluded;
- Samples with at least one missing value in our reanalysis results for peptide validation were excluded;
- After application of the first two exclusion criteria, group “type IIa” had only three samples. Since this group was too small for the training of a machine learning-based model, the whole group was excluded from validation;
- Finally, samples with a weakly predominant MYH isoform and that were outliers in the principal component analysis were excluded.

As a result, a total of 26 samples were excluded due to the above criteria. The remaining validation data set includes 29 samples and three groups of muscle fibers: 9 in group “type I”, 12 in group “T2b”, and 8 in group “type IIx”. The following table shows all 29 samples from Murgia et al. that were included in the validation data set.

**Table A1:** Samples in the validation data set. In this table, all 29 samples that were included in the validation data set for the identified two-peptide panel for the discrimination of muscle fiber types are listed. In the column “Original sample ID”, the original sample IDs used by Murgia et al. and for the raw files in PRIDE are shown. In the column “Sample ID within this work”, the standardized sample IDs used within this work are shown.

Sample	Original Sample ID	Sample ID Within This Work
1	1107fib_E34	Type IIb_2
2	1107fib_S1	Type I_1
3	Fib_1011E26	Type IIx_1
4	Fib_1011E8a	Type IIx_2
5	Fib_1011E8b	Type IIx_3
6	Fib_1011Sol2	Type I_2
7	Fib_1011Sol3	Type IIx_4
8	SA_0430_e10	Type IIb_4
9	SA_0430_e5	Type IIb_5
10	SA_0430_S11	Type I_3
11	SA_1211E111	Type IIb_7
12	SA_1211E112	Type IIb_8
13	SA_1211E92	Type IIx_6
14	SA_1211S15	Type I_4
15	SA_24Ott_25E	Type IIb_9
16	SA_24Ott_e14	Type IIb_10
17	SA_fib_0108_EDL1_2	Type IIb_11
18	SA_fib_0108_EDL2_1	Type IIx_8
19	SA_fib_0108_EDL2_2	Type IIx_9
20	SA_Fib_0716_E13_130718153850	Type IIb_12
21	SA_Fib_0716_E9	Type IIb_13
22	SA_fib_0716_S61	Type I_5
23	SA_fib_0716_S62	Type I_6
24	SA_fib_0719_14E	Type IIb_14
25	SA_fib_0719_18E	Type IIb_15
26	SA_fib_0719_S1	Type I_7
27	SA_Fib_1128_E10	Type IIx_10
28	SA_Fib_1128_S21	Type I_8
29	SA_Fib0114_S8	Type I_10
