# Supplementary material for: Advanced Fiber Type-Specific Protein Profiles Derived from Adult Murine Skeletal Muscle
Source: Proteomes. 2021 Jun 8;9(2):28. doi: 10.3390/proteomes9020028 (PMC8293376; doi:10.3390/proteomes9020028)
Supplement: Supplementary file 1 [file proteomes-09-00028-s001.zip › Supplementary_Figure_Description.pdf]

Figure S1: Myosin (MYH) Isoform Staining: a) Soleus muscle stained with BA-F8 specific for MYH7 (type I fibers). b) Soleus muscle stained with SC-71 specific for MYH2 (type IIa fibers). c) Tibialis anterior muscle stained with BF-F3 specific for MYH4 (type IIb fibers). d) Tibialis anterior muscle stained with 6H1 specific for MYH1 (type IIx fibers). All antibodies were purchased from the Developmental Studies Hybridoma Bank (DSHB) (Iowa City, Iowa).

Figure S2: GO term enrichment analysis (Cellular compartment (CC)) of cluster members of cluster 1. GO terms were sorted chronologically. Dot sizes indicate fold enrichment values and color intensities correspond to corrected p-values (Benjamini-Hochberg). Highest fold enrichment scores are sorted by rank (Rank Fold Enrichment).

Figure S3: GO term enrichment analysis (Cellular compartment (CC)) of cluster members of cluster 2. GO terms were sorted chronologically. Dot sizes indicate fold enrichment values and color intensities correspond to corrected p-values (Benjamini-Hochberg). Highest fold enrichment scores are sorted by rank (Rank Fold Enrichment).

Figure S4: GO term enrichment analysis (Cellular compartment (CC)) of cluster members of cluster 3. GO terms were sorted chronologically. Dot sizes indicate fold enrichment values and color intensities correspond to corrected p-values (Benjamini-Hochberg). Highest fold enrichment scores are sorted by rank (Rank Fold Enrichment).

Figure S5: GO term enrichment analysis (Cellular compartment (CC)) of cluster members of cluster 4. GO terms were sorted chronologically. Dot sizes indicate fold enrichment values and color intensities correspond to corrected p-values (Benjamini-Hochberg). Highest fold enrichment scores are sorted by rank (Rank Fold Enrichment).

Figure S6: Pathway enrichment analysis of cluster members of cluster 1. Pathway terms were sorted alphabetically. Dot sizes indicate fold enrichment values and color intensities correspond to corrected p-values (Benjamini-Hochberg). Highest fold enrichment scores are sorted by rank (Rank Fold Enrichment).

Figure S7: Pathway enrichment analysis of cluster members of cluster 2. Pathway terms were sorted alphabetically. Dot sizes indicate fold enrichment values and color intensities correspond to corrected p-values (Benjamini-Hochberg). Highest fold enrichment scores were sorted by rank (Rank Fold Enrichment).

Figure S8: Pathway enrichment analysis of cluster members of cluster 3. Pathway terms were sorted alphabetically. Dot sizes indicate fold enrichment values and color intensities correspond to corrected p-values (Benjamini-Hochberg). Highest fold enrichment scores are sorted by rank (Rank Fold Enrichment).

Figure S9: Pathway enrichment analysis of cluster members of cluster 4. Pathway terms were sorted alphabetically. Dot sizes indicate fold enrichment values and color intensities correspond to corrected p-values (Benjamini-Hochberg). Highest fold enrichment scores are sorted by rank (Rank Fold Enrichment).

Figure S10: Decision tree for the classification of all sub fiber types. The root node of the decision tree contains 100% of the data of all sub fiber type groups. The alpha-actinin 3 peptide TINEVENQVLTR is taken to test the first decision rule. Here it is verified whether the log LFQ

value of this peptide is < 19. For 27% of all samples, this rule does not apply and they are sorted into a terminal node (= leaf node) and are classified as type IIb fibers. The remaining 73% are tested using the second classification rule, verifying whether the log LFQ value of the myosin-1 peptide IAEQELLDASER is < 22. This is true for all type I fiber type samples (27% of the samples) and they get sorted into the leaf node to the left and are thus completely classified. For all other samples, the third decision rule is applied, in which type II and type IIx fibers are distinguished applying a log LFQ threshold for the TINEVENQVLTR peptide. As a result, all samples are classified correctly.

Figure S11: a,b) Boxplots displaying the intensity value distributions on a log2-scale of all assessed fiber types from the public dataset. Peptide IAEQELLDASER is discriminating type I and type IIb from type IIx fibers (a) and peptide TINEVENQVLTR is discriminating type I from type IIb and type IIx fibers (b). c) Decision tree for the classification of the different fiber types from the public data set. The first classification rule (log LFQ values > 24 for alpha-actinin-3 peptide TINEVENQVLTR) is able to determine type I fibers. Samples with log LFQ values < 24 are classified with the second decision rule (log LFQ value < 28 for myosin-1 peptide IAEQELLDASER) into either type IIb or type IIx fibers.
