# Supplementary figures and images for: Advanced Fiber Type-Specific Protein Profiles Derived from Adult Murine Skeletal Muscle
Source: Proteomes. 2021 Jun 8;9(2):28. doi: 10.3390/proteomes9020028 (PMC8293376; doi:10.3390/proteomes9020028)

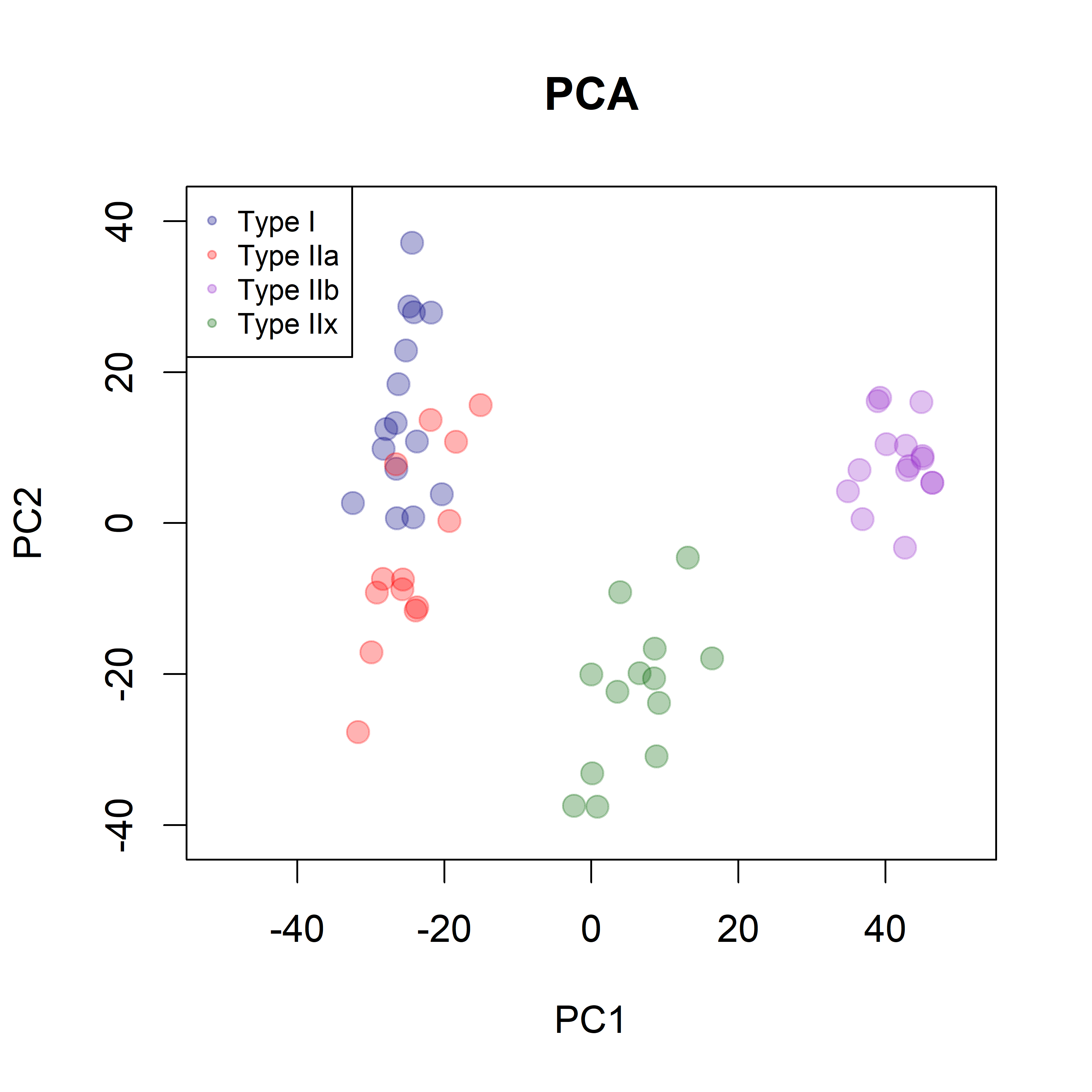

Supplement: Supplementary file 1 [file proteomes-09-00028-s001.zip › FiPSPi/FiPSPi_results/01_pca_1_2_filteredFeatures.png]

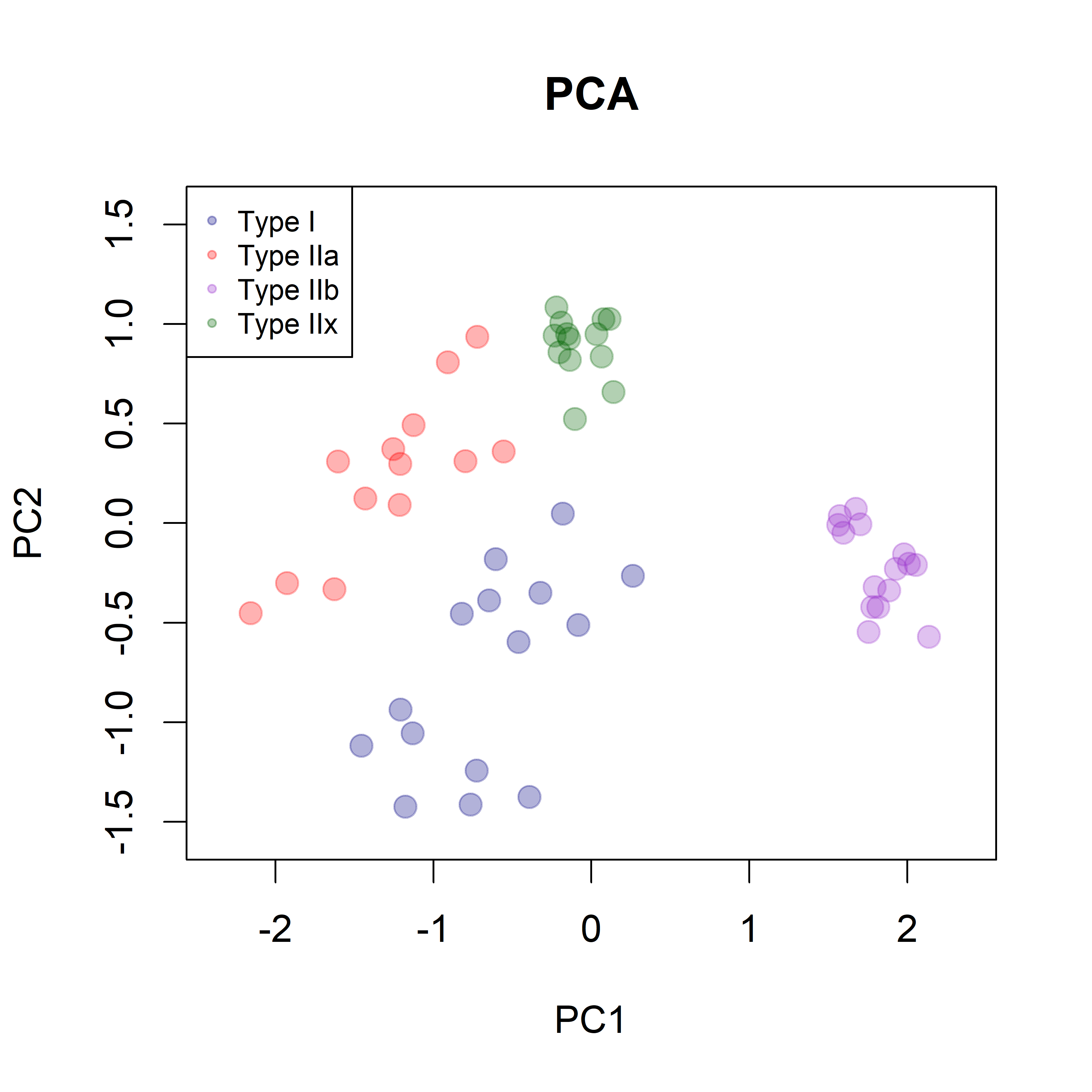

Supplement: Supplementary file 1 [file proteomes-09-00028-s001.zip › FiPSPi/FiPSPi_results/01_pca_1_2_selectedFeatures.png]

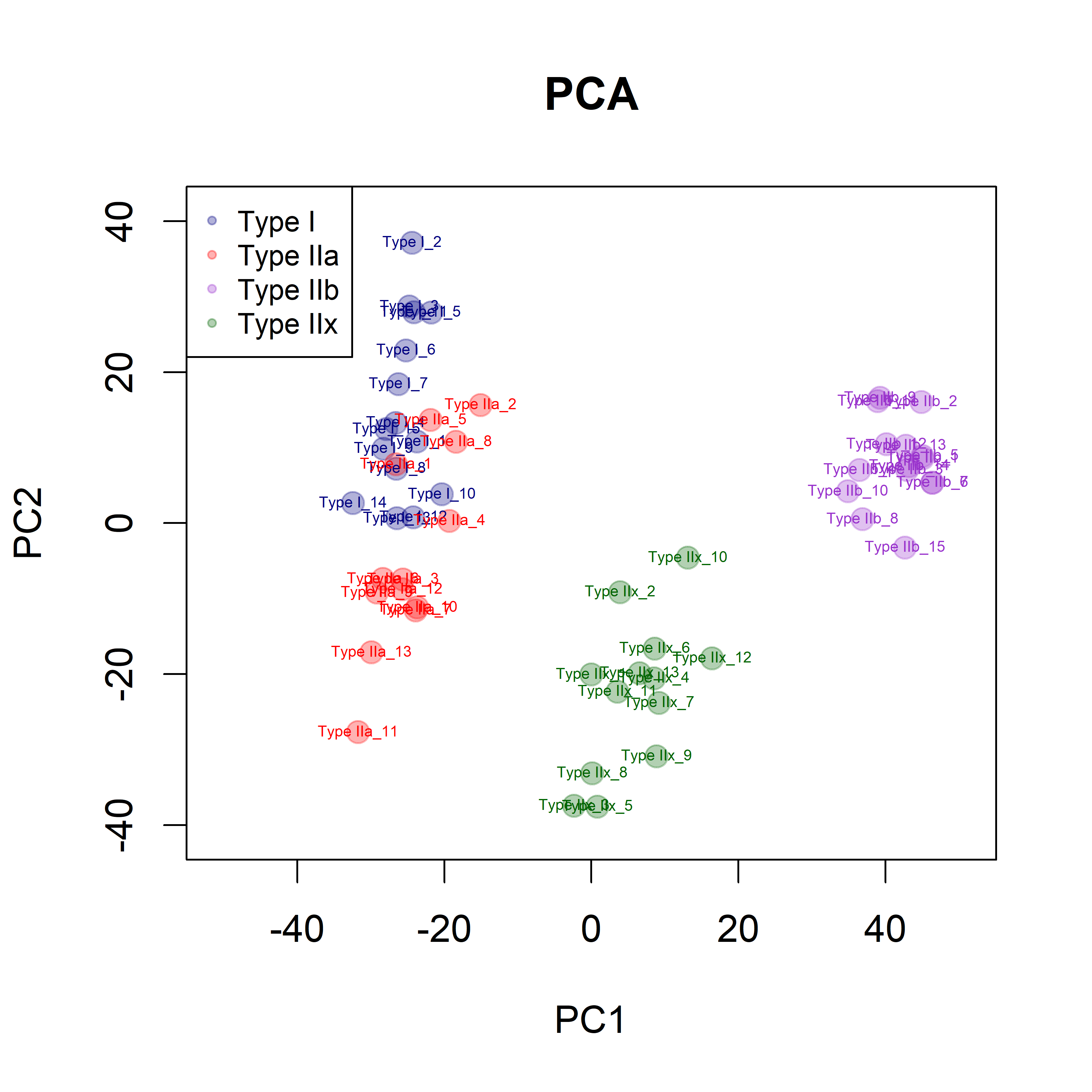

Supplement: Supplementary file 1 [file proteomes-09-00028-s001.zip › FiPSPi/FiPSPi_results/02_pca_1_2_filteredFeatures.png]

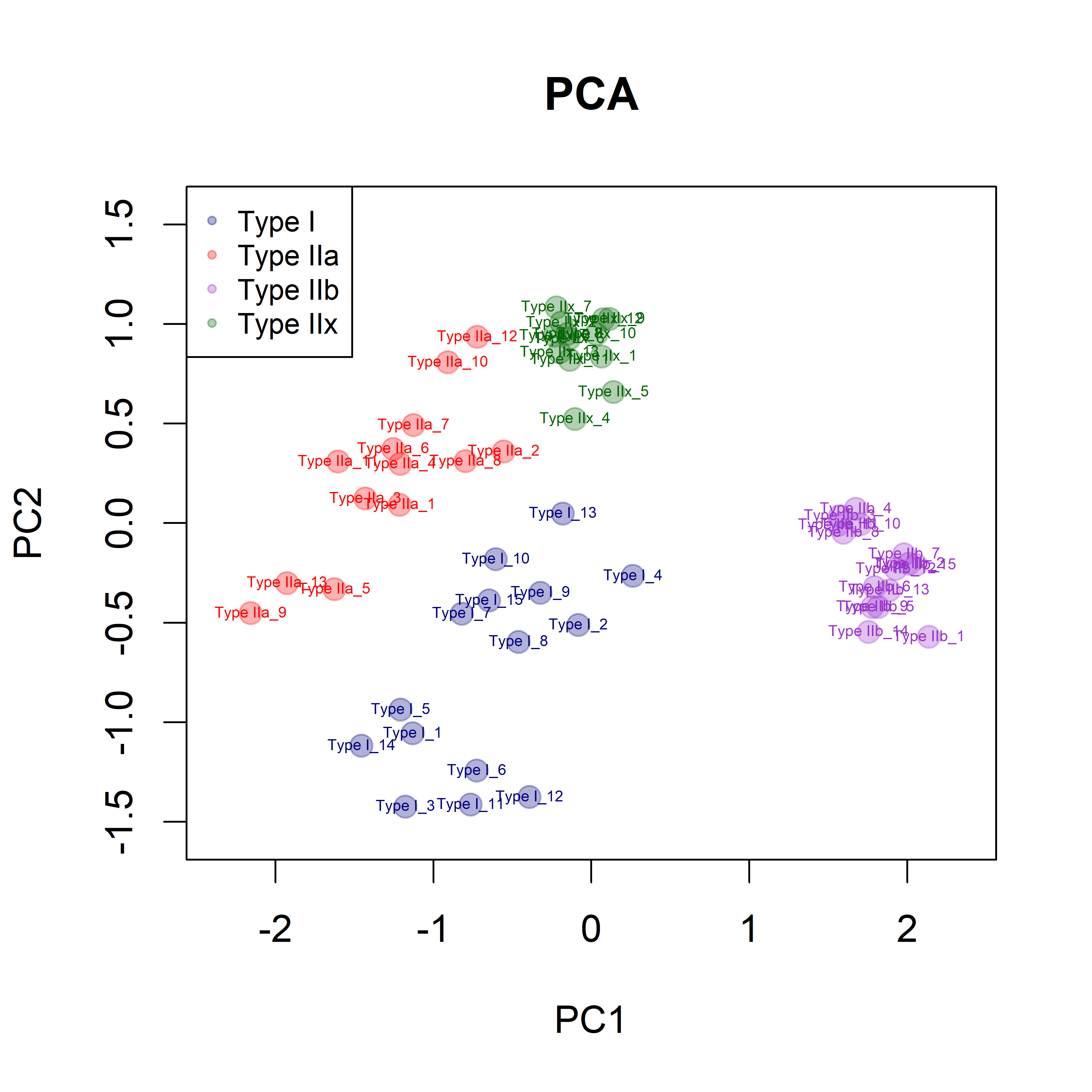

Supplement: Supplementary file 1 [file proteomes-09-00028-s001.zip › FiPSPi/FiPSPi_results/02_pca_1_2_selectedFeatures.png]

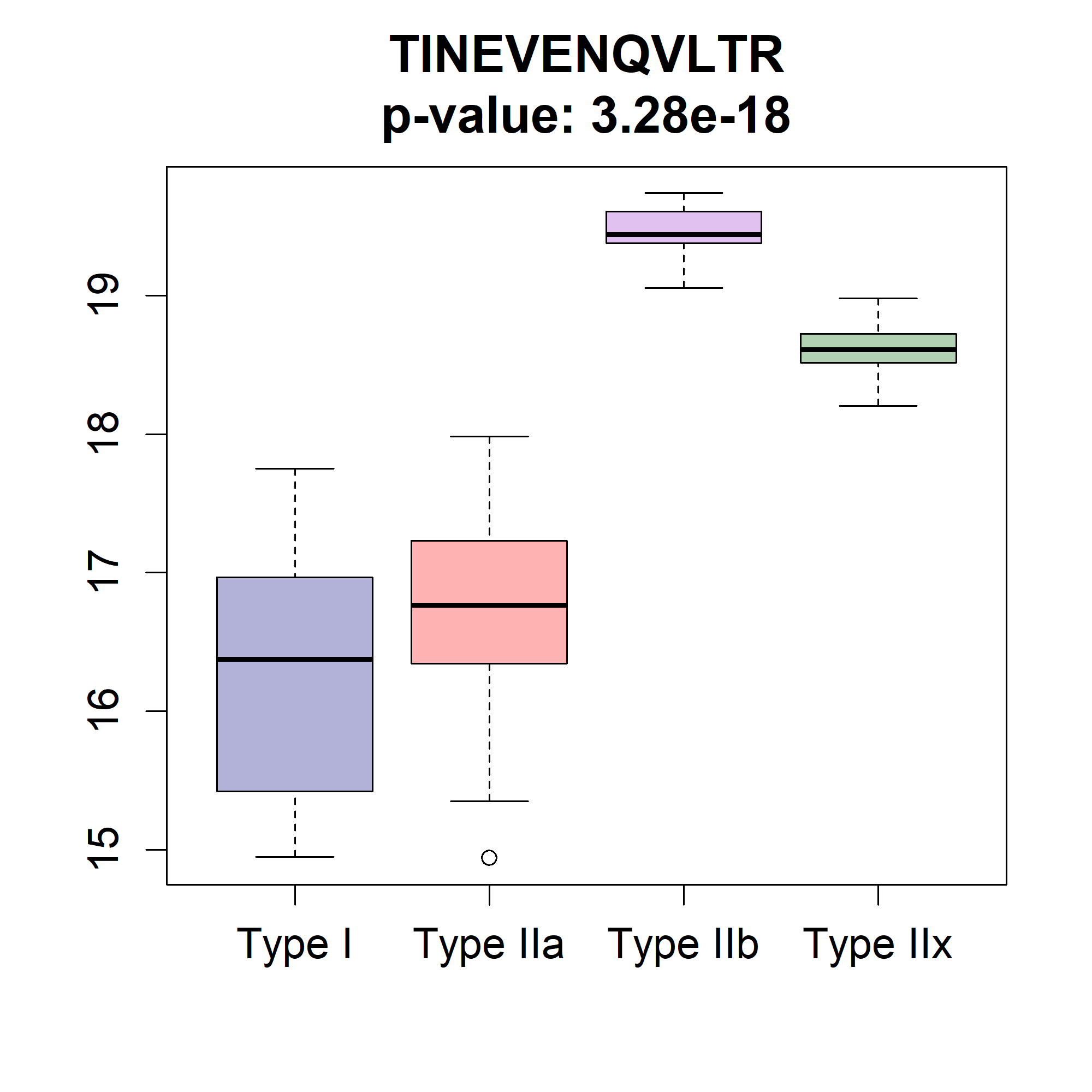

Supplement: Supplementary file 1 [file proteomes-09-00028-s001.zip › FiPSPi/FiPSPi_results/boxplot_1_O88990_peptide38.png]

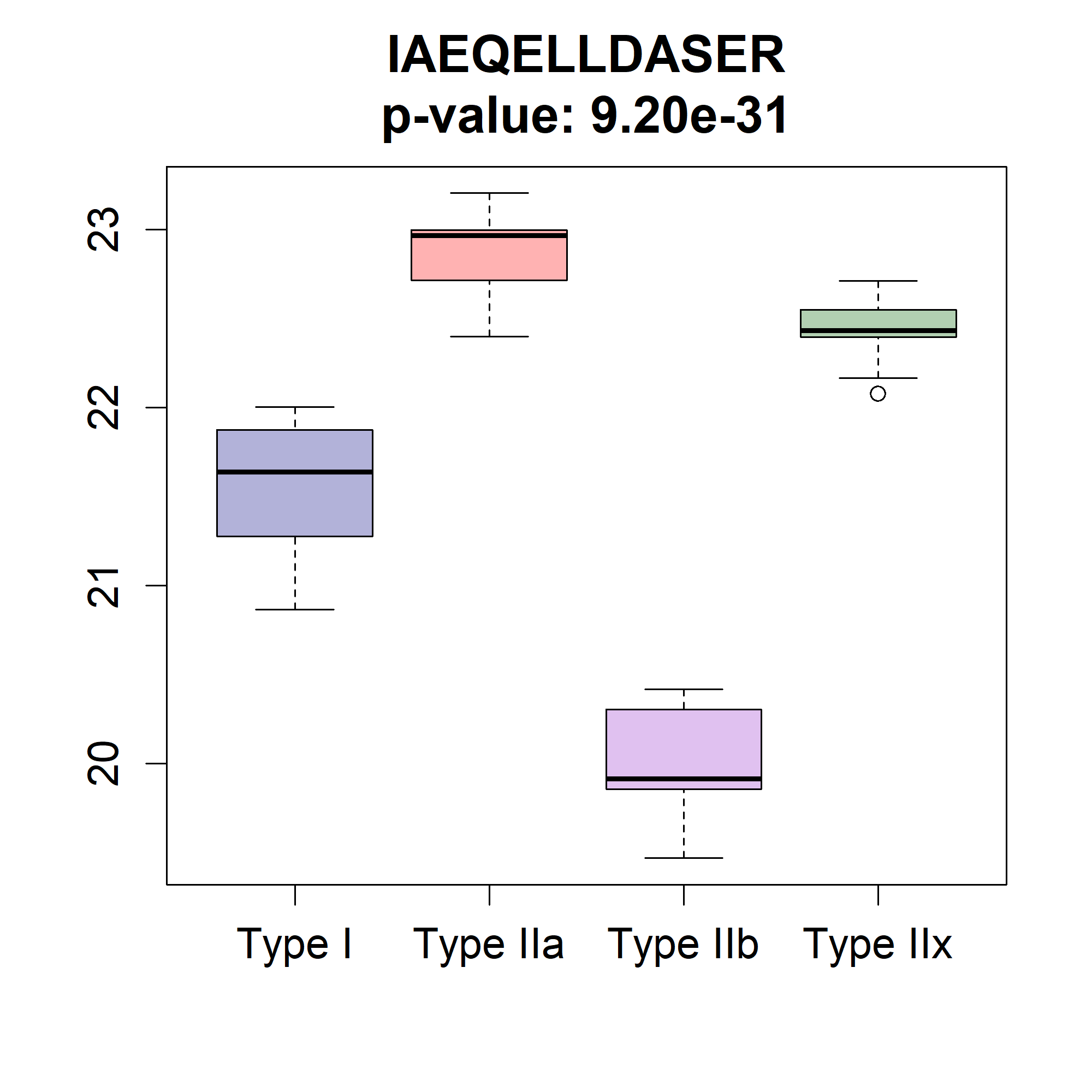

Supplement: Supplementary file 1 [file proteomes-09-00028-s001.zip › FiPSPi/FiPSPi_results/boxplot_2_Q5SX40_peptide40.png]

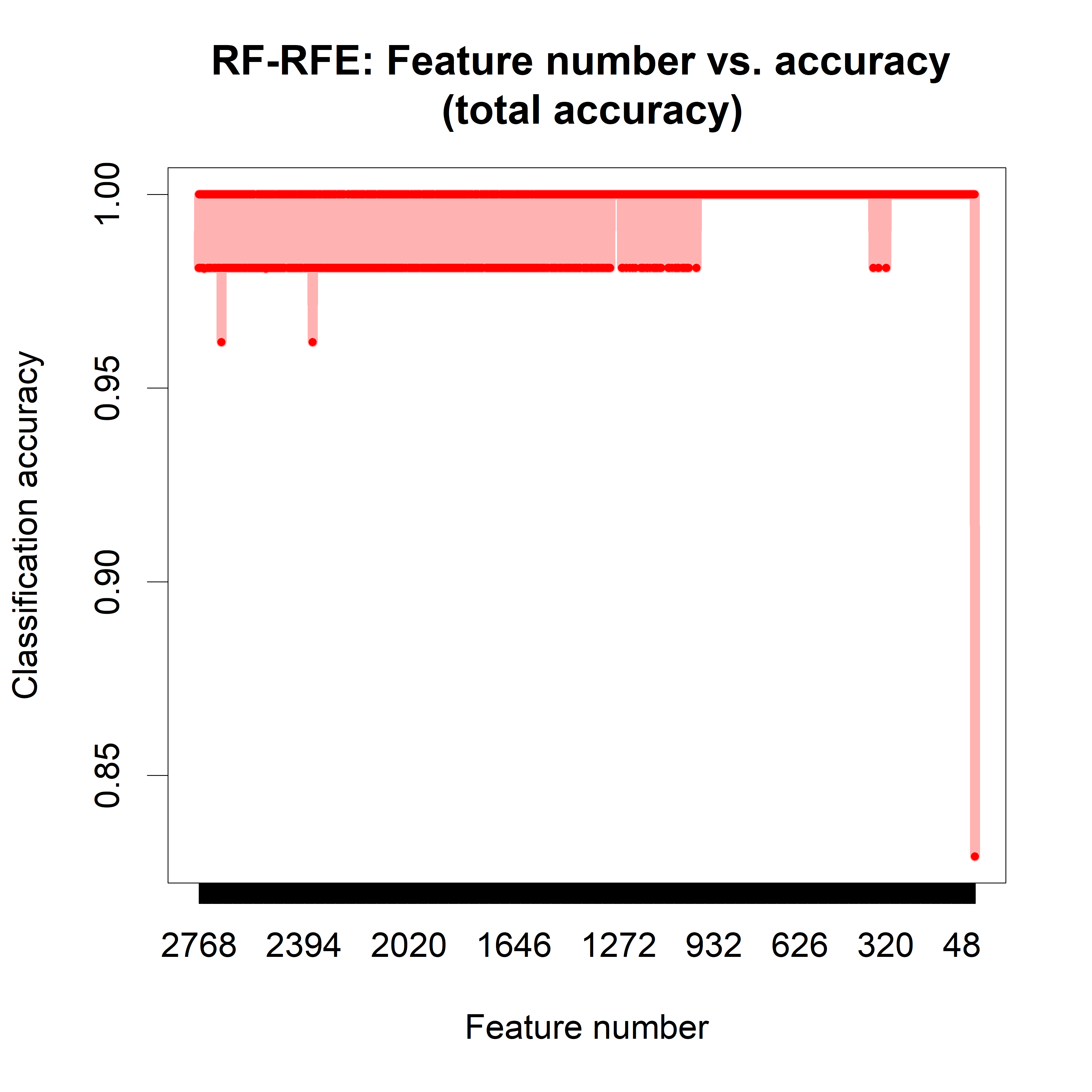

Supplement: Supplementary file 1 [file proteomes-09-00028-s001.zip › FiPSPi/FiPSPi_results/rf-rfe_featNumber_vs_accuracy.png]

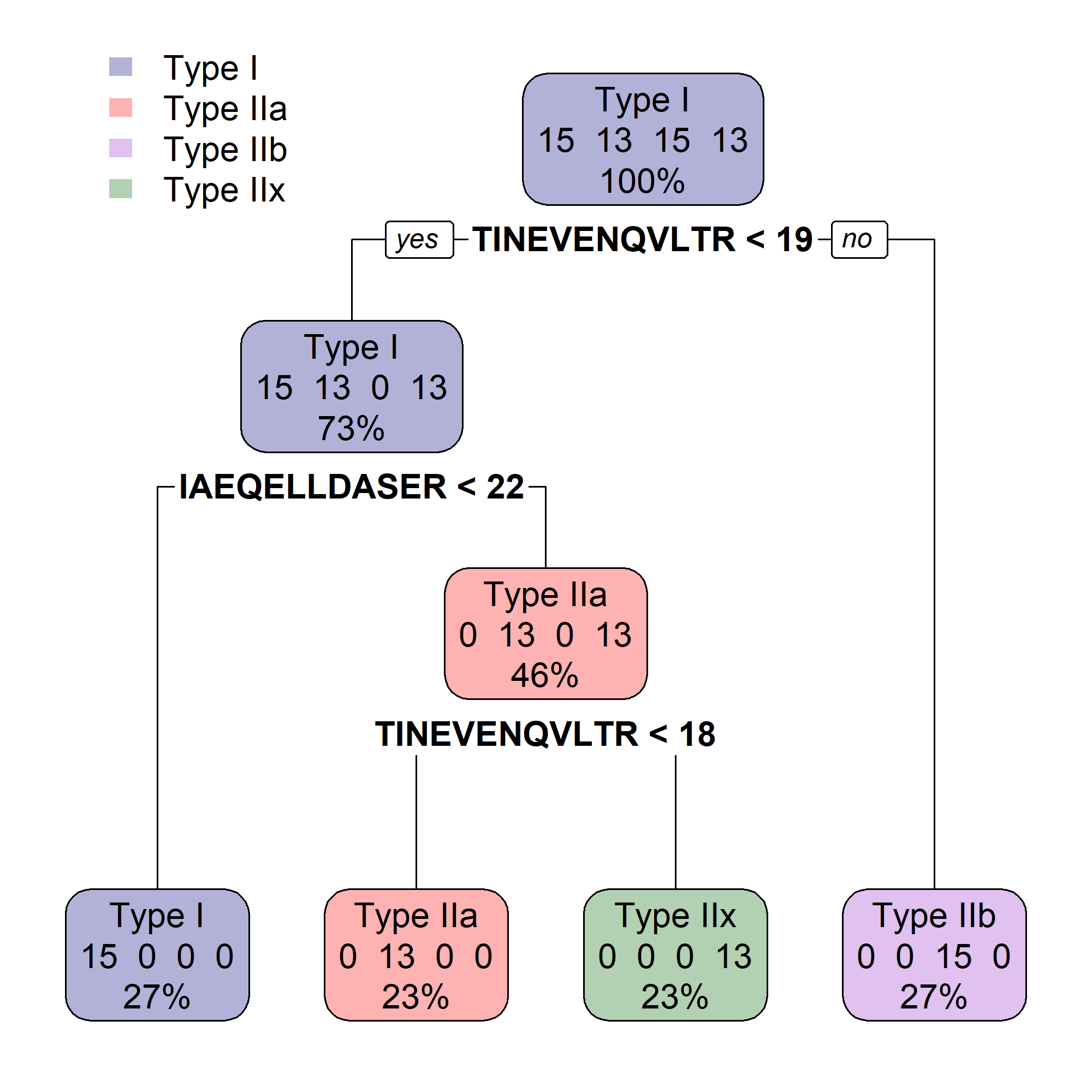

Supplement: Supplementary file 1 [file proteomes-09-00028-s001.zip › FiPSPi/FiPSPi_results/rpart-plot.png]

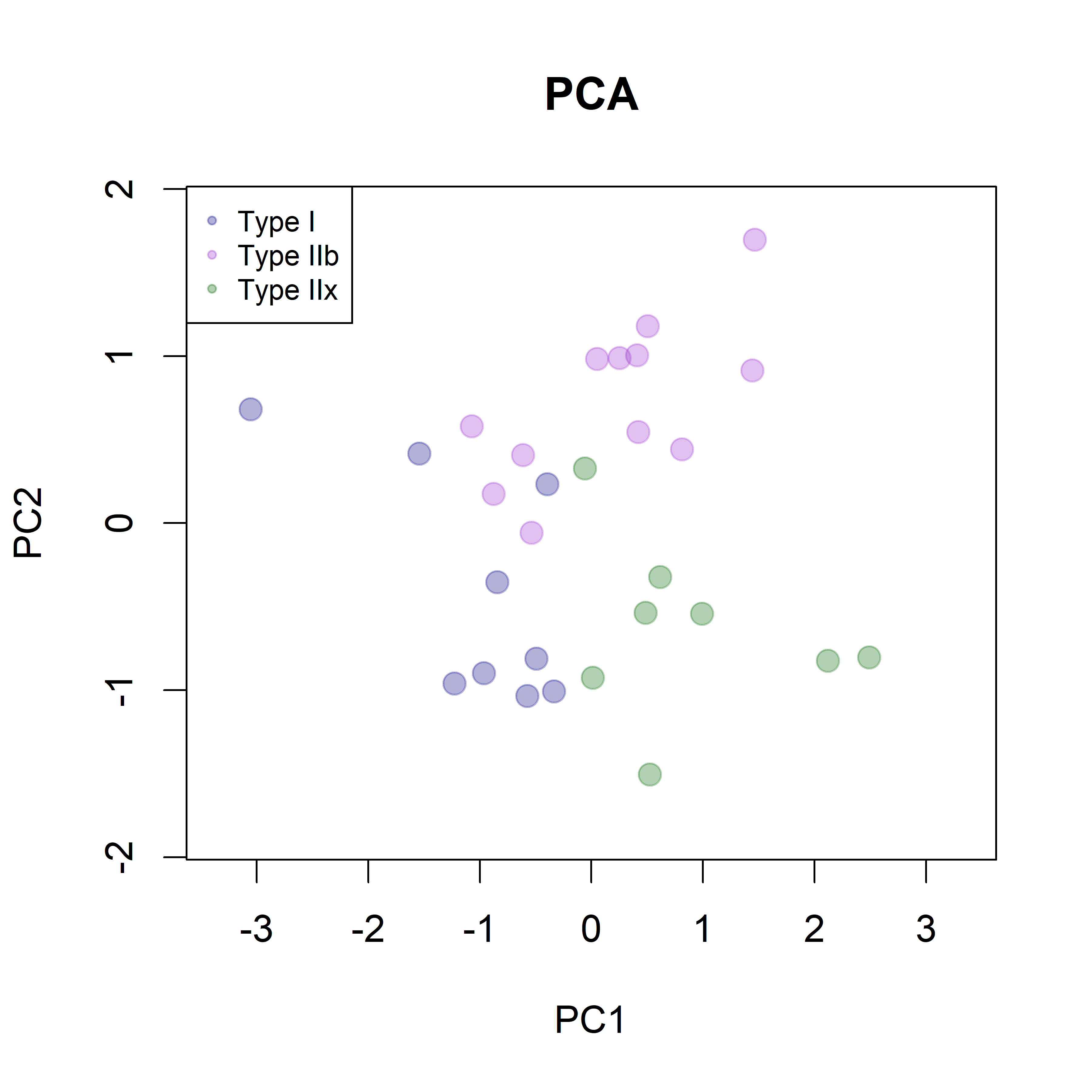

Supplement: Supplementary file 1 [file proteomes-09-00028-s001.zip › FiPSPi/FiPSPi-validation_results/01_pca_1_2_selectedFeatures.png]

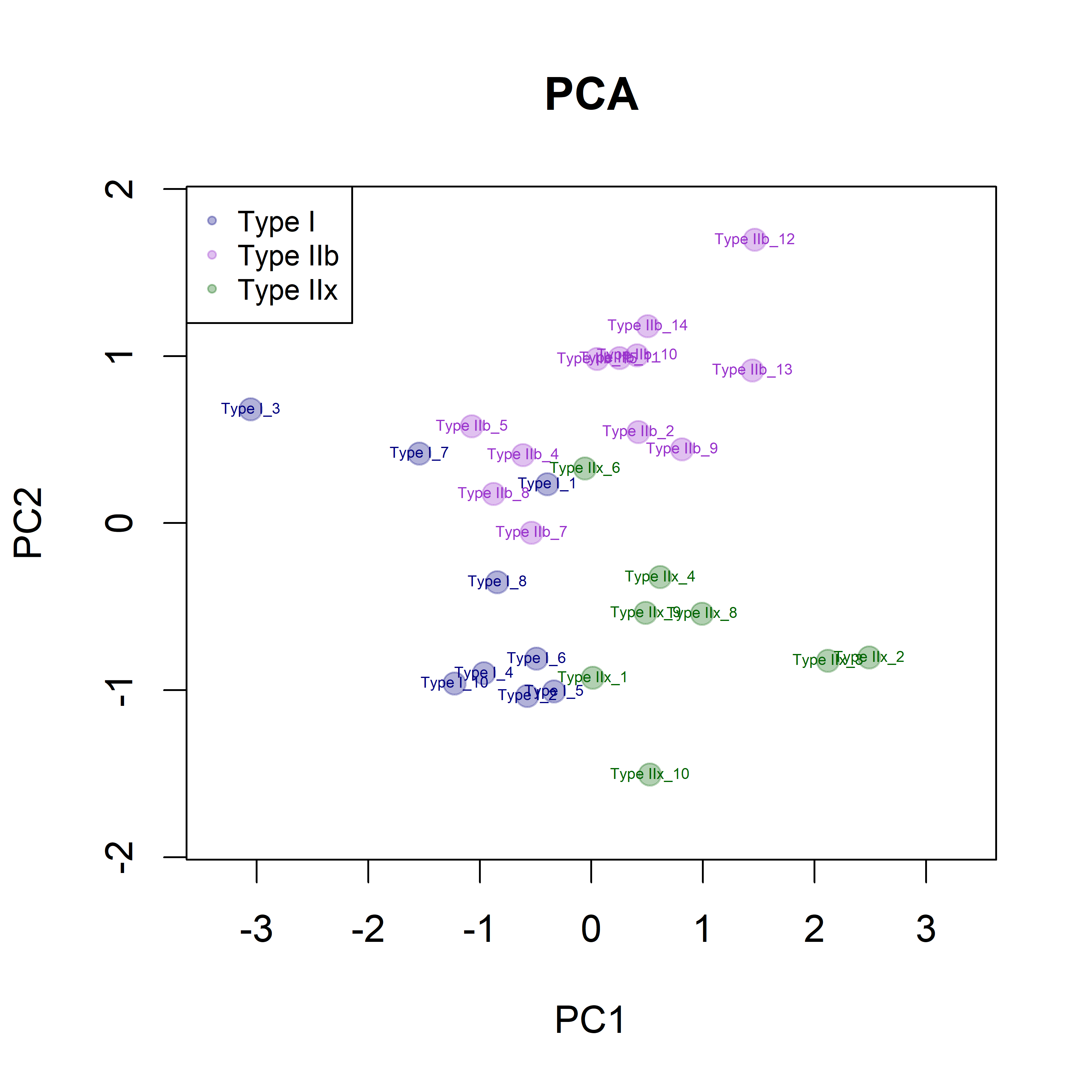

Supplement: Supplementary file 1 [file proteomes-09-00028-s001.zip › FiPSPi/FiPSPi-validation_results/02_pca_1_2_selectedFeatures.png]

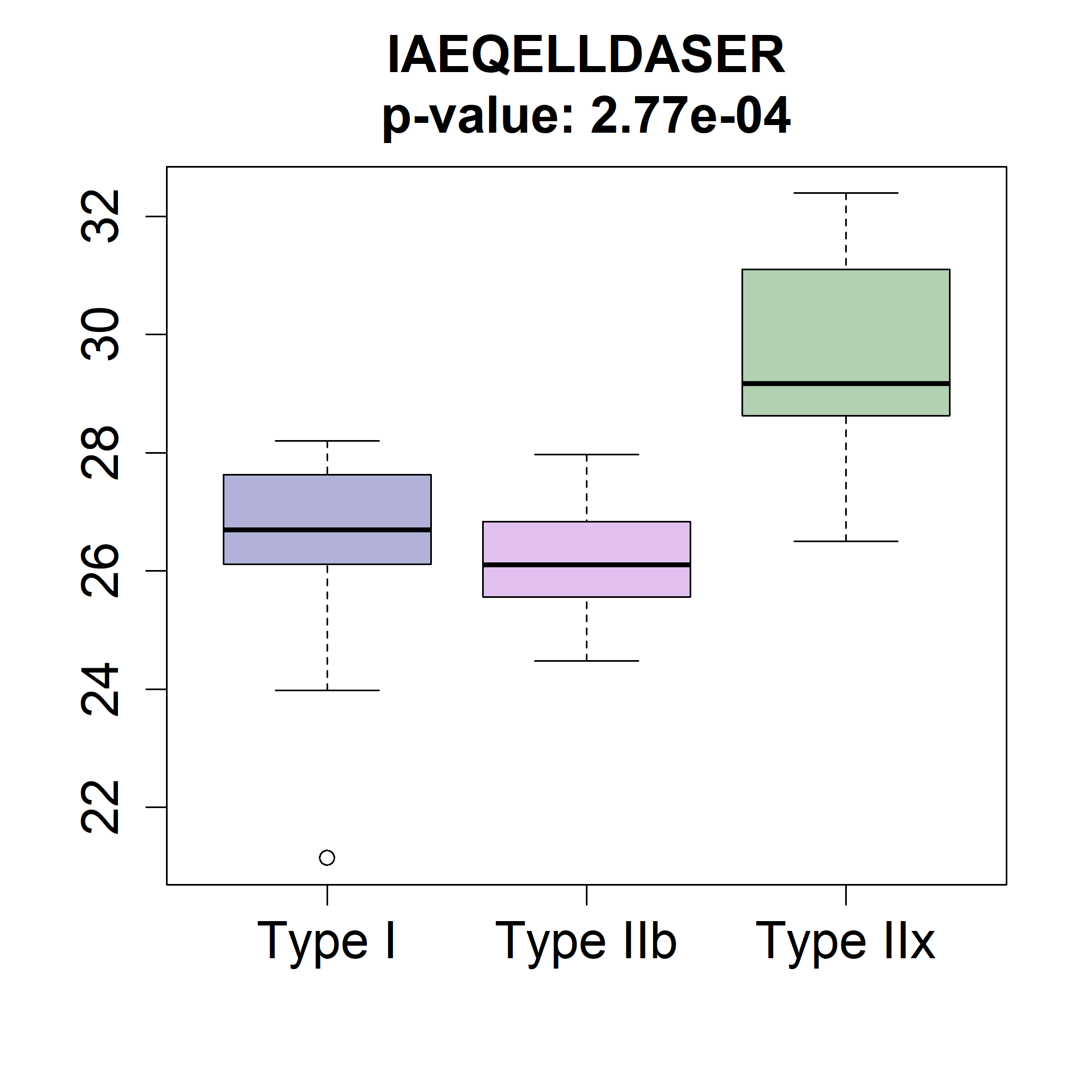

Supplement: Supplementary file 1 [file proteomes-09-00028-s001.zip › FiPSPi/FiPSPi-validation_results/boxplot_1_TINEVENQVLTR.png]

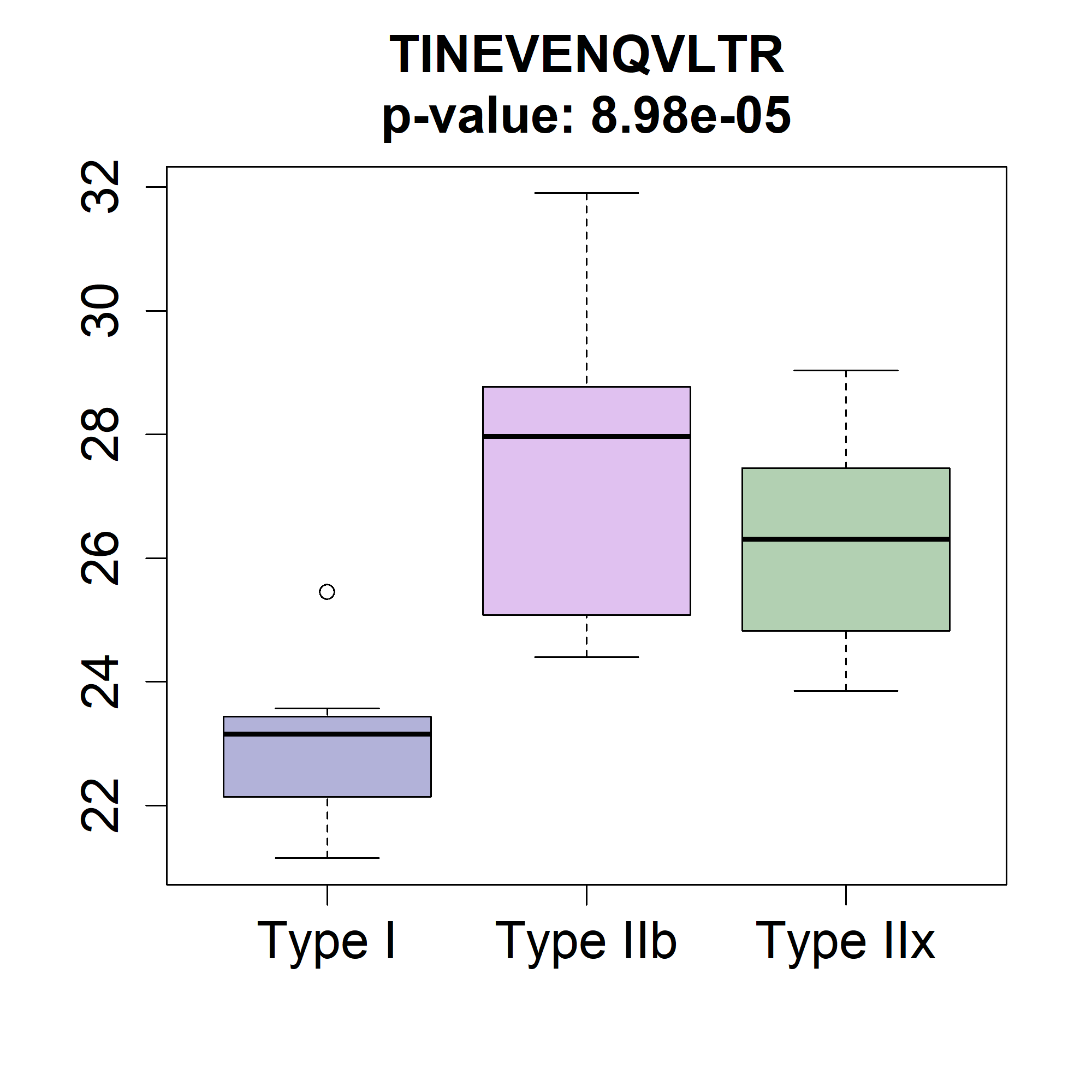

Supplement: Supplementary file 1 [file proteomes-09-00028-s001.zip › FiPSPi/FiPSPi-validation_results/boxplot_2_IAEQELLDASER.png]

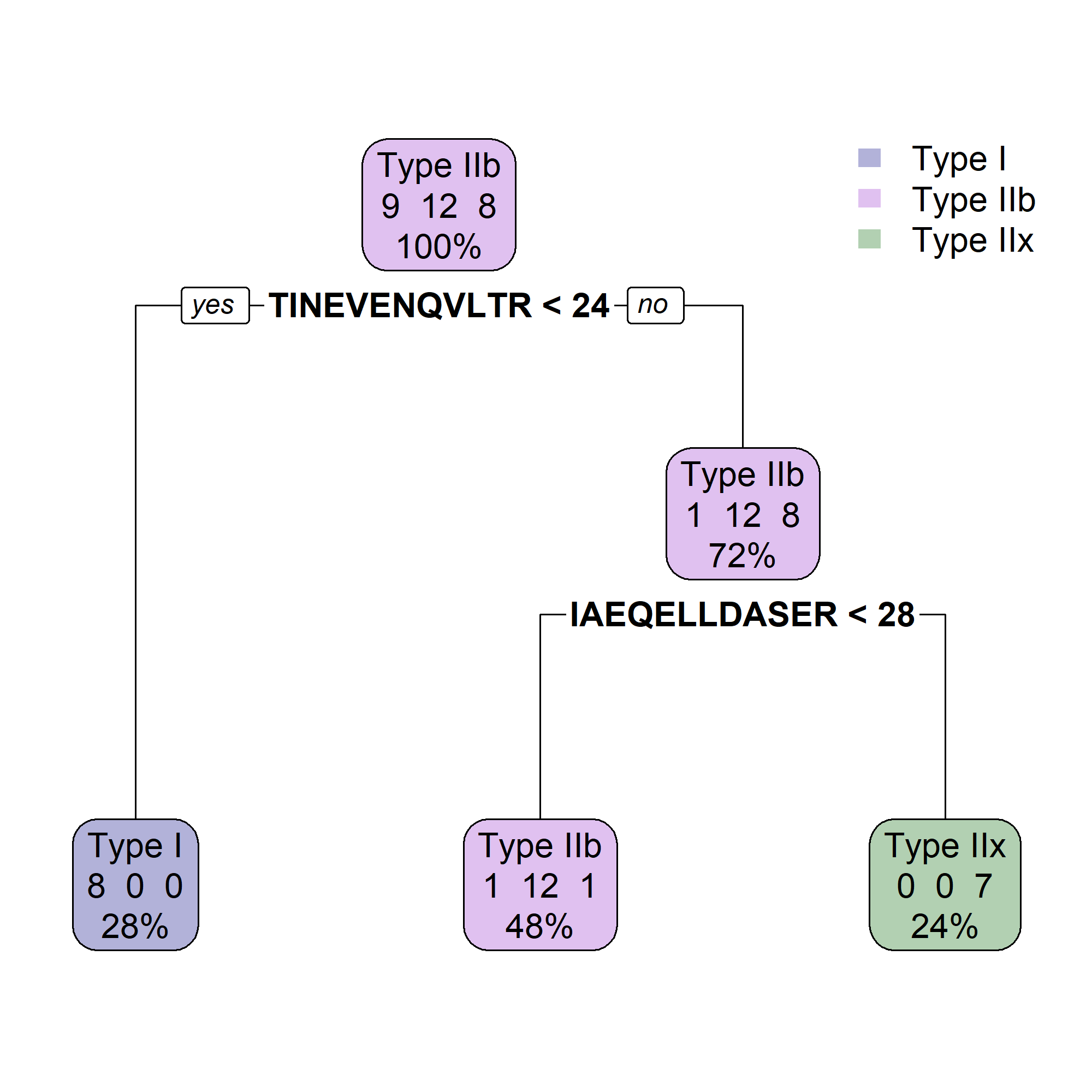

Supplement: Supplementary file 1 [file proteomes-09-00028-s001.zip › FiPSPi/FiPSPi-validation_results/rpart-plot.png]

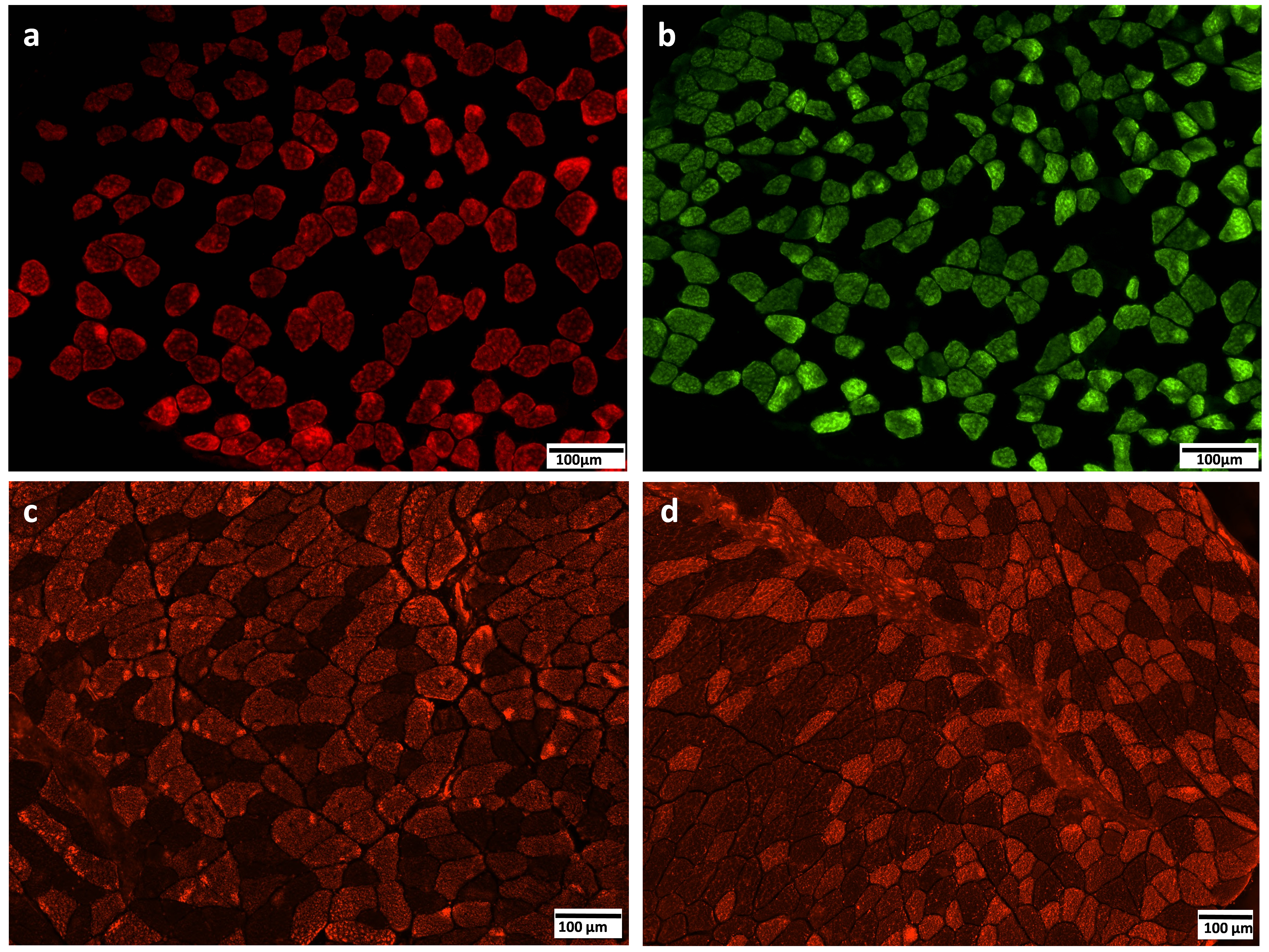

Supplement: Supplementary file 1 [file proteomes-09-00028-s001.zip › Supplementary figures/S1_MYH_Isoform_Staining.tif]

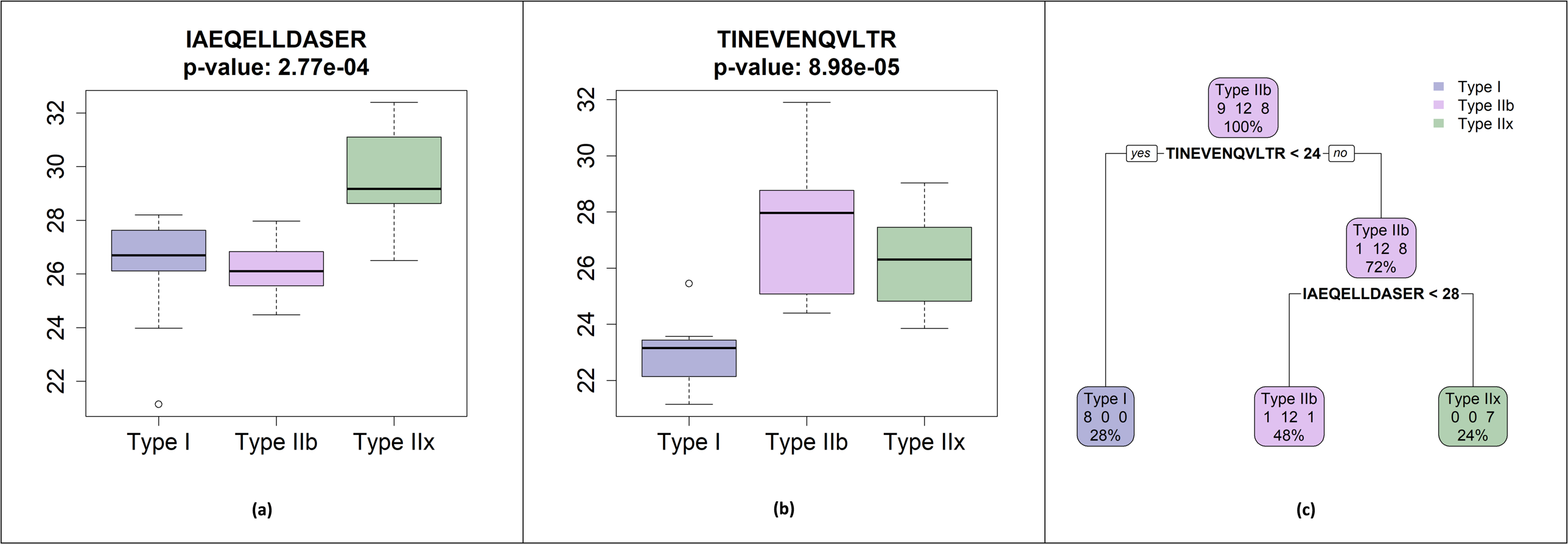

Supplement: Supplementary file 1 [file proteomes-09-00028-s001.zip › Supplementary figures/S11_Public_Dataset_Samples.tif]

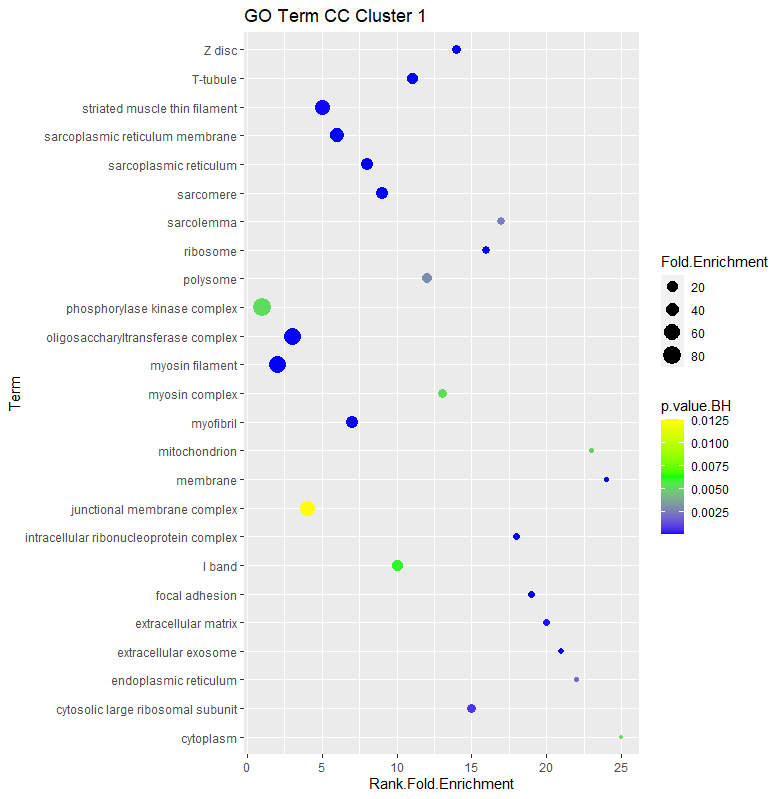

Supplement: Supplementary file 1 [file proteomes-09-00028-s001.zip › Supplementary figures/S2_Bubbleplot_GO Term CC Cluster 1.png]

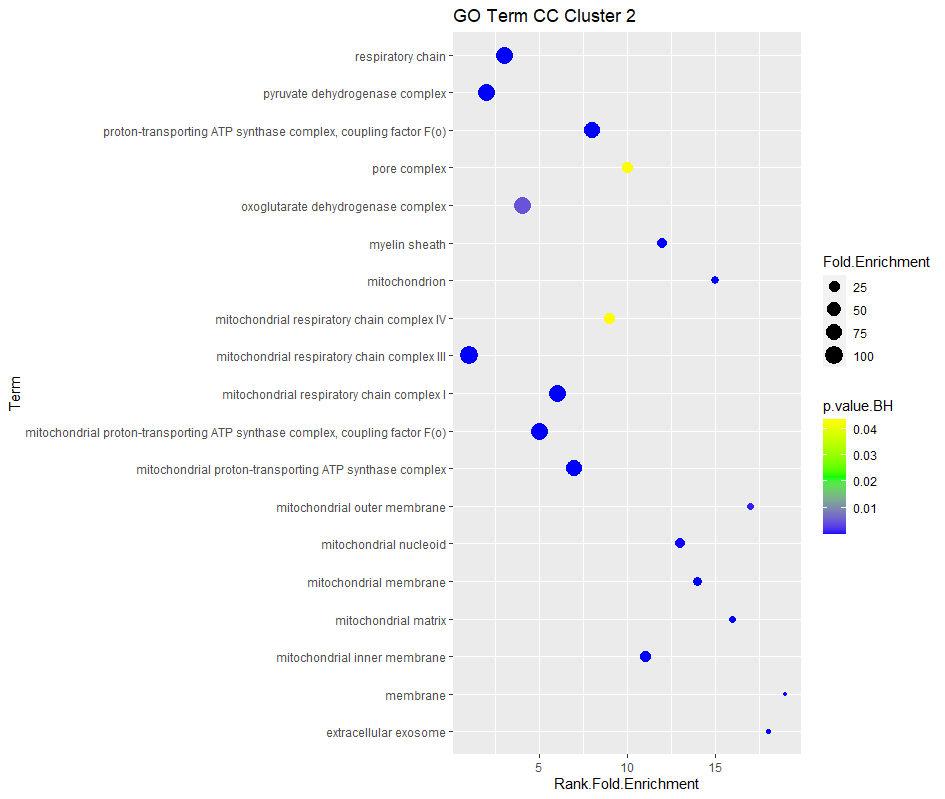

Supplement: Supplementary file 1 [file proteomes-09-00028-s001.zip › Supplementary figures/S3_Bubbleplot_ GO Term CC Cluster 2.png]

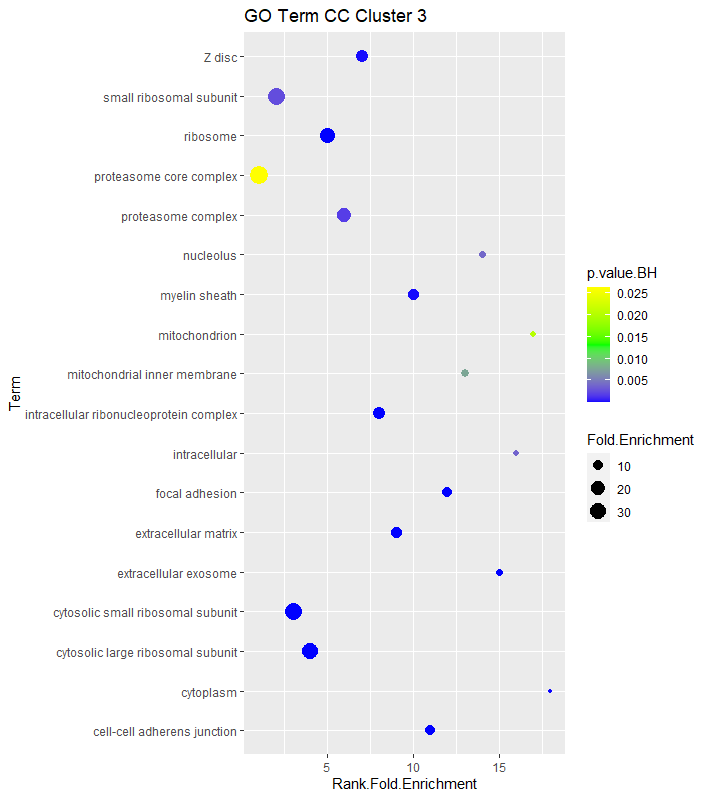

Supplement: Supplementary file 1 [file proteomes-09-00028-s001.zip › Supplementary figures/S4_Bubbleplot_ GO Term CC Cluster 3.png]

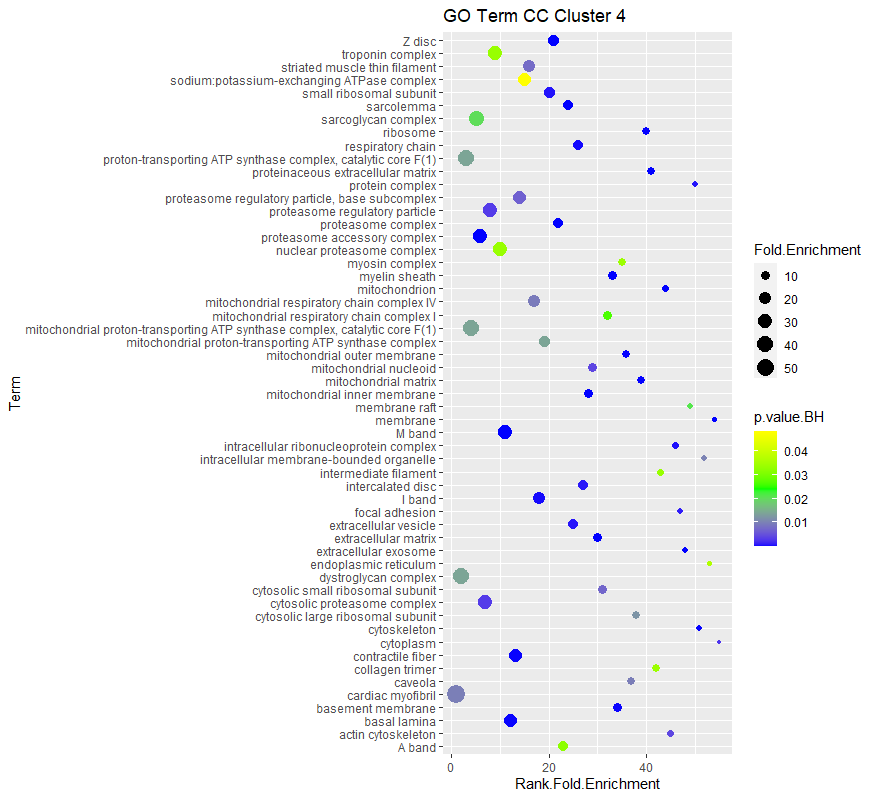

Supplement: Supplementary file 1 [file proteomes-09-00028-s001.zip › Supplementary figures/S5_Bubbleplot_GO_Term CC Cluster 4.png]

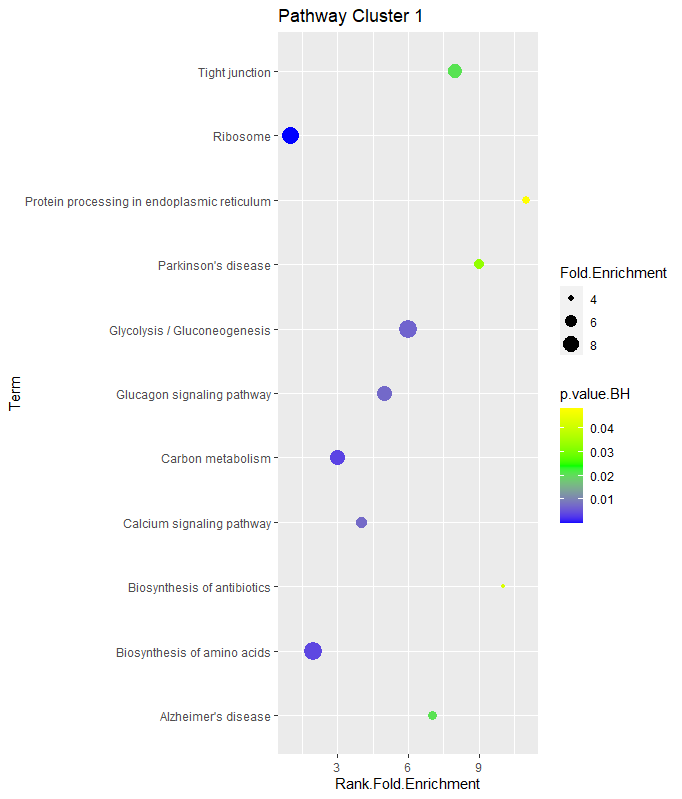

Supplement: Supplementary file 1 [file proteomes-09-00028-s001.zip › Supplementary figures/S6_Bubbleplot_Pathway Cluster 1.png]

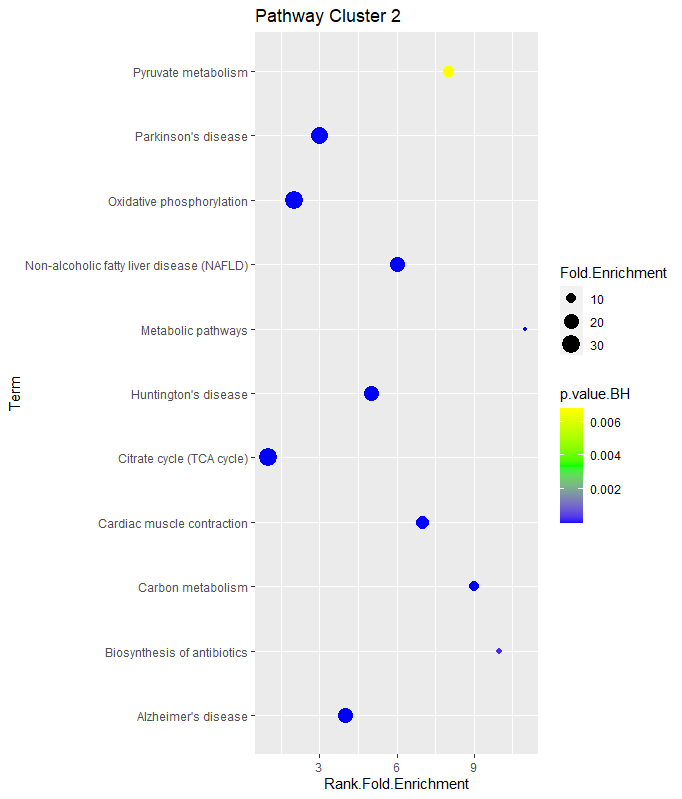

Supplement: Supplementary file 1 [file proteomes-09-00028-s001.zip › Supplementary figures/S7_Bubbleplotplot_Pathway Cluster 2.png]

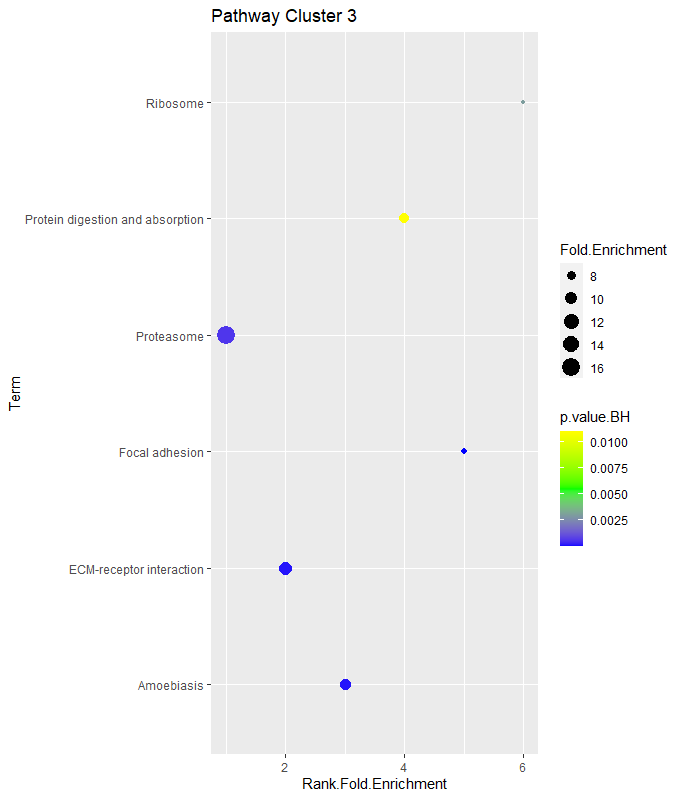

Supplement: Supplementary file 1 [file proteomes-09-00028-s001.zip › Supplementary figures/S8_Bubbleplotplot_Pathway Cluster 3.png]

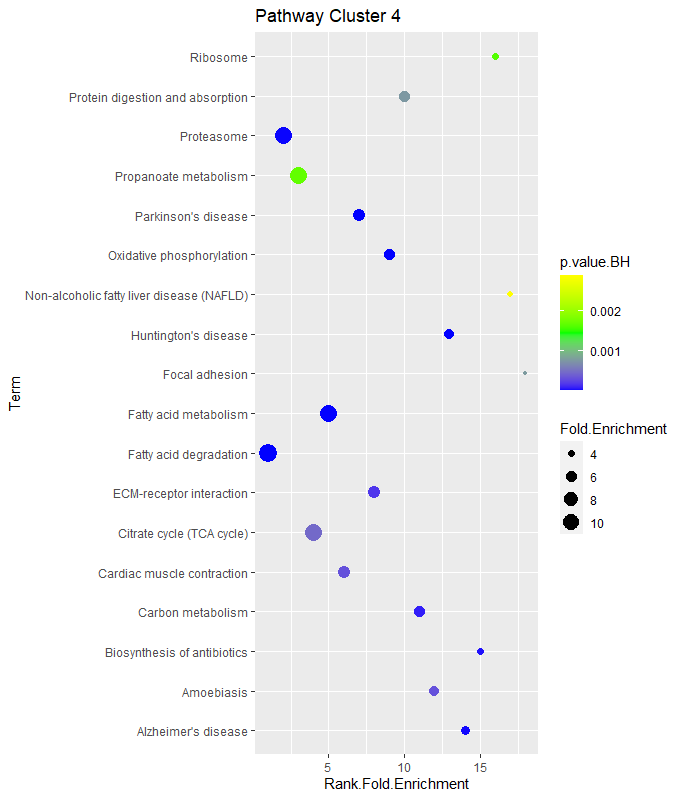

Supplement: Supplementary file 1 [file proteomes-09-00028-s001.zip › Supplementary figures/S9_Bubbleplotplot_Pathway Cluster 4.png]
